# Network-based approach highlighting interplay among anti-hypertensives: target coding-genes: diseases

**DOI:** 10.1038/s41598-020-76605-1

**Published:** 2020-11-19

**Authors:** Reetu Sharma

**Affiliations:** Centre for Molecular Modeling, CSIR-Indian Institute of Chemical Technology, Tarnaka, Hyderabad India

**Keywords:** Molecular medicine, Hypertension

## Abstract

Elucidating the relation between the medicines: targets, targets: diseases and diseases: diseases are of fundamental significance as-is for societal benefit. Hypertension is one of the dangerous health conditions prevalent in society, is a risk factor for several other diseases if left untreated and anti-hypertensives (AHs) are the approved drugs to treat it. The goal of the study is to decipher the connection between hypertension with other health conditions, however, is challenging due to the large interactome. To fulfill the aim, the strategy involves prior clustering of the AHs into groups as per our previous method, followed by the analyzing functional association of the target coding-genes (tc-genes) and health conditions for each group. Following our recently published work where the AHs are clustered into six groups such that molecules having similar patterns come together, here, the distribution of molecular functions and the cellular components adopted by the tc-genes of each group are analyzed. The analyses indicate that kidney, heart, brain or lung related ailments are commonly associated with the tc-genes. The association of selective tc-genes to health conditions suggests a preference for certain health conditions despite many possibilities. Analyses of experimentally validated drug–drug combinations indicate the trend in successful AHs combinations. Clinically validated combinations bind different targets. Our study provides a promising methodology in a network-based approach that considers the influence of structural diversity of AHs to the functional perspective of tc-genes concerning the health conditions. The method could be extended to explore disease–disease relationships.

## Introduction

The drug–target interaction is intrinsically associated with a disease process to produce a desired therapeutic effect by enhancing or inhibiting a function^1^. The binding of a molecule to its target is chiefly governed by the spatial arrangement of structural frameworks^2^. The concept of one drug–one disease–one target is failing as the human functional interactome involves multi-targets in complex sub-networks and is transitioning towards network mode with multi-drugs: multi-targets: multi-diseases concept^3–5^. The symbol “:” describes the association in the paper. It is a challenge to decipher the disease mechanisms of the drug–target interactions owing to the complexity of interpreting the proteomics network in disease^3^. Considering the vast data related to human target coding-genes (tc-genes) are available, retrieving meaningful information in a novel way for easy interpretation of the interactome based outlook is needed^6^.

Network based approaches have been recently popular in unravelling diverse objectives^7^; for example, in the prediction of drug–drug combinations^8^, to quantify the disease–disease^9^, drug–disease relationships^10^, drug efficacy screening^11^, and repurposing^5^, therefore serve as a valuable tool to follow the cell’s functional organization^12^. Disease gene products are likely to cluster in a same network neighborhood. The tc-genes and the drugs represent nodes within molecular networks often are coupled in both therapeutic and adverse effects. The network based functional analyses of a health condition can be used to identify the trend and commonalities associated with it^10^.

One such common and dangerous health condition prevalent in today’s generation is hypertension. It is characterized by persistent elevated blood pressure, ordinarily displays no symptoms^13^. If not treated, it may ultimately lead to other severe health issues, thus needs explicit attention^14^. Patients failing to achieve adequate reductions in blood pressure through DASH (Dietary Approaches to Stop Hypertension) are prescribed anti-hypertensives (AHs) by physicians^15^. Hydralazine^16^ and minoxidil^17^ (vasodilating drugs); hydrochlorothiazide^18^ and chlorthalidone^18^ (thiazide diuretics); atenolol^19^ and metoprolol (angiotensin-converting enzyme inhibitors); amlodipine^18^ and nifedipine^20^ (calcium channel blockers); valsartan, telmisartan, olmesartan and losartan^21^ (angiotensin II receptor blockers) are a few of the first-line drugs that have been generic for several years. With the vast amount of AHs and genome-wide association study (GWAS) of tc-genes available, an explicit, comprehensive investigation that could relate the tc-genes and the subsequent diseases is interesting in its own right.

Several recent investigations to interpret the mechanism of AHs action are reported. Wu et al.^22^ suggests a mechanism of *Uncaria* alkaloids treating hypertension through a network based approach that includes protein–protein interaction network, topology analysis and molecular docking. Sharma^23^ devised a three-tiered unsupervised learning approach to cluster the molecules into minimum groups such that significant molecules displaying similar predominant patterns comes together with minimal human intervention. Principal component analysis and k-means followed by statistical check using one-way analysis of similarities (ANOSIM) allow the selection of the final cluster^23^. The strategy clusters the AHs into six groups; a brief introduction about the pattern of each one is as follows; group 1 (g1) includes the molecules with total four–six rings, of which two–three rings are five-membered; molecules possessing two–three rings and all are six-membered belong to group 2 (g2), the most populated one; group 3 (g3) involves AHs having two rings, of which at least one is a five-membered ring; group 4 (g4) consists of molecules comprising four–six rings, where the ratio of total to six-membered rings is less than 1.4; group 5 (g5) molecules consist of three-membered rings, of which two are six-membered and the third one is either a five or seven-membered ring system; lastly, the monocyclic molecules constitute group 6 (g6)^23^.

The interactome-based network, although is visually appealing, often provides little information due to its complexity^24,25^. Extending our efforts in unravelling the trends associated with the medicines or diseases^23,25–27^, in this paper, we investigate the functional and structural patterns of AHs that have been clustered into six groups in our previous publication^23^ with the tc-genes and diseases. Clustering of AHs based on common structural patterns prior to analyzing the functional association of tc-genes and diseases allow simplifying the large-scale network into sub-networks. Altogether, this strategy provides insights into the relation of hypertension to other diseases. Here, the trends associated with successful drug–drug combinations and functional aspects of the popular marketed AHs have been extracted. The present work provides a powerful methodology to analyze AHs: tc-genes: diseases relationship that can be applied to elucidate other disease–disease relationships.

## Materials and methods

### Data mining

U.S. Food and drug administration agency (FDA) approved anti-hypertensives (AHs) were searched from DrugBank (v5.1.2)^28^ leaving molecules in the clinical trials, experimental, investigational or withdrawn phase drugs as of December 2018. The dataset is composed of 114 AHs obtained from DrugBank. The details of the AHs considered for the study are in Table S1. The structural information about the desired medicines was retrieved from DrugBank. Any of the six groups is referred as group x (gx); for example g1, g2, etc. The tc-genesx is the tc-genes associated with a gx; for example, tc-genes1 refers to the tc-genes associated with g1 and so forth.

### Constructing AHs: tc-genes interactome network

The targets and the corresponding tc-genes information extracted from the UniProt Knowledgebase (UniProtKB)^29^ were retained if the following criteria were followed: (i) the target must be from homo sapiens, (ii) the target should be represented by unique UniProt accession, and (iii) the target status need to be marked as reviewed and the bioactivity data for the selected drug–target pairs, collected from ChEMBL (v20, accessed in December 2018)^30^ shows inhibition constant/potency (K_i_), dissociation constant (K_d_) or median effective concentration (EC_50_) ≤ 10 μM. The information retrieved was visualized using Cytoscape (v2.8.3)^31^, an open source software platform. The gx, tx and tc-genesx were represented as nodes. The edges represented the undefined network’s interaction. The common genes among the groups were demonstrated in the form of a matrix.

The percentage of common tc-genes between tc-genesx and tc-genesy (C_g;_ %);

$$C_{g} = \frac{{n\left( {{\rm{tc}} - {\rm{genesx }} \cap {\rm{ tc}} - {\rm{genesy}}} \right)}}{{n\left( {{\rm{tc}} - {\rm{genesx }} \cup {\rm{ tc}} - {\rm{genesy}}} \right)}} \times 100$$

$$n\left(\rm{tc}-\rm{genesx }\cup \rm{ tc}-\rm{genesy}\right)=n\left(\rm{tc}-\rm{genesx}\right)+ n\left(\rm{tc}-\rm{genesy}\right)-n\left(\rm{tc}-\rm{genesx }\cap \rm{ tc}-\rm{genesy}\right)$$

### Gene ontology (GO)

The functional information about the tc-genes was inferred using GO annotation, retrieved from UniProtKB^29^. Experimentally validated or evidence supported literature for the molecular functions (MF) and cellular components (CC) were extracted excluding the computationally inferred annotations. MF and CC denote the molecular activities and the components where the target is reported as active, respectively. Redundancy in the GO was removed before performing the functional analyses. MF activities having frequency of occurrence (FOc) more than two in a group were considered for evaluation, except for the g1 where FOc of two is considered acceptable owing to its smallest group size.

### Pairwise combinations of AHs

The gold-standard pairwise combinations were collected by assembling clinical data as mentioned in Cheng et al.^8^. The generic name of AHs was standardized by MeSH vocabularies^32,33^. Each drug in combination should have experimentally validated target and must follow the criteria mentioned in “Constructing AHs: tc-genes interactome network” section. The clinically successful combinations were assembled from multiple sources. The duplicates were removed and 21 combinations of AHs were selected for consideration in the study (Table S5).

### tc-genes: disease association

The relevance (p-value) indicating the association between the tc-genes and diseases with p-value ≤ 2.0E−9 was extracted from an open source database called Open Targets (OT)^34^. 2.0E−9 is the maximum p-value present in all the six groups, hence, was considered as a threshold. The threshold value is chosen such that each group OT platform integrates evidence to a target using Ensembl stable IDs^35^ and the association between diseases by mapping them to experimental factor ontology (EFO) terms^34^. OT uses human GWAS data for systematic drug target identification and prioritization.

The association score (s) between tc-gene and diseases;

$${\rm{s }} = {\rm{ F }}*{\rm{ S }}*{\rm{ C}}$$

F is the relative occurrence of tc-gene and disease evidence. S is the magnitude or strength of the effect described by the evidence. C is the overall confidence for the observation that generates the tc-gene and disease evidence.

The definition of diseases is as according to the OT database. Hereafter, the word “diseases” and “health conditions” are used interchangeably. The health conditions relating to a tc-gene were distributed according to decreasing relevance (p-value). The p-value has an inverse relation to the associated health condition. Lower the p-value, higher is the possibility that the health condition is not associated with the tc-gene by chance. Top 20 health conditions (according to minimum p-value) were analyzed for each group’s distribution and tc-gene: disease network analyses.

## Results and discussion

The objective of this section is as follows; (i) identification of the unique tc-genes associated with the AHs and determining the prevalence of MF activity of the tc-genes, (ii) demonstrating the distribution of CC, (iii) network-based analysis of tc-genes in relation to AHs, (iv) analyzing the common tc-genes, (v) the link of the tc-genes with other diseases, and (vi) examining the clinically validated drug–drug combinations. The tc-genes associated with each group are listed in Table 1.

**Table 1:** The number of molecules and the tc-genes belonging to each group.

S. no	Group number	No. of molecules	Unique associated genes (tc-genes)
1	g1	08	07
2	g2	32	60
3	g3	24	44
4	g4	12	31
5	g5	14	46
6	g6	24	16

### GO: MF analyses

**Figure 1:**
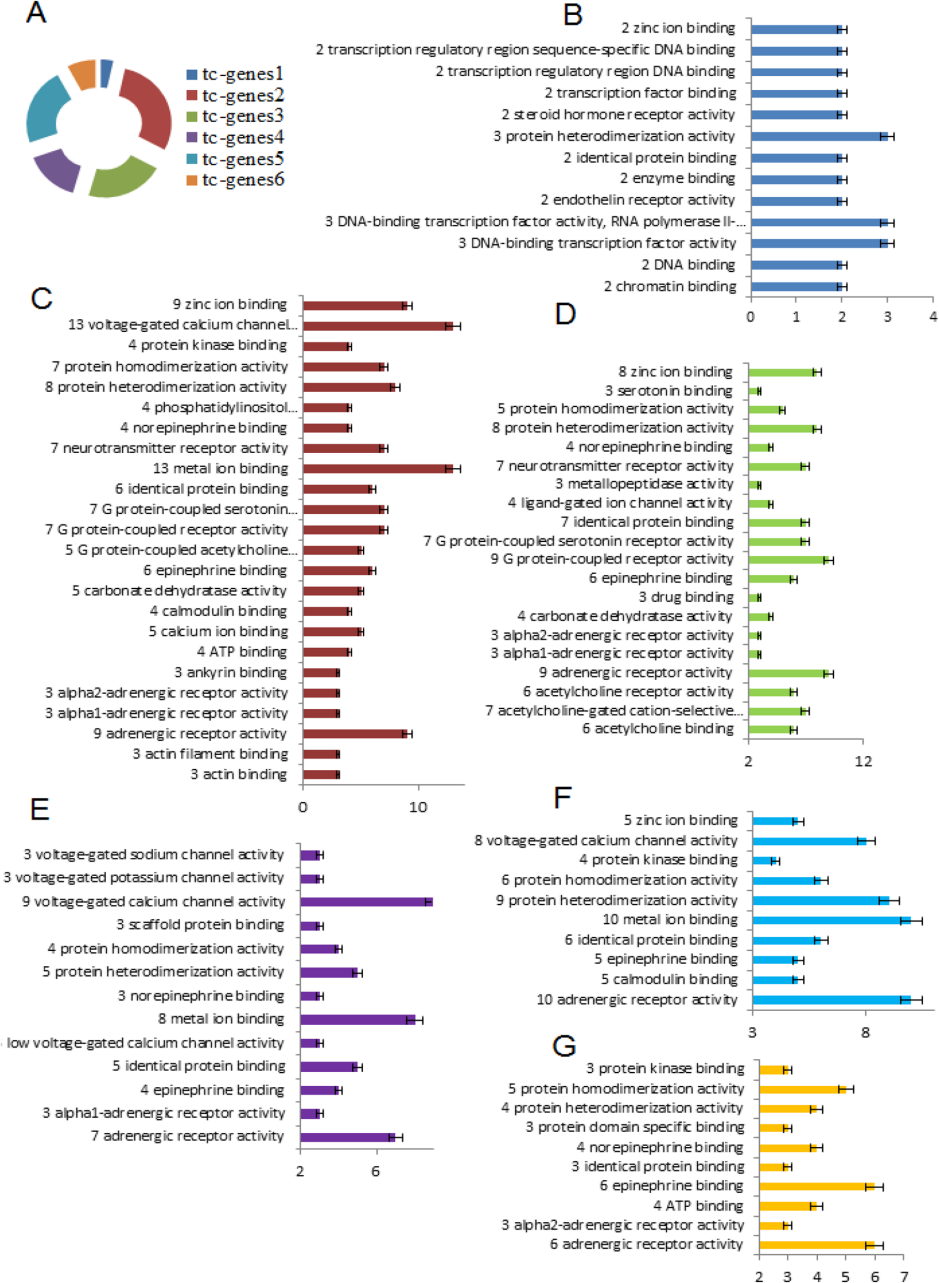
GO: MF analyses. (**A**) The percentage of unique tc-genes associated with each group, (**B**) tc-genes1: dark blue, (**C**) tc-genes2: maroon, (**D**) tc-genes3: green, (**E**) tc-genes4: violet, (**F**) tc-genes5: light blue, and (**G**) tc-genes6: orange, respectively. The number of instances of each MF is written at the beginning of the MF name. The MF (y-axis) activities associated with the tc-genes (x-axis) are represented in (**B**–**G)**. The error associated with each column represents 5% of the total value.

The g1 has fewest fractions of the tc-genes associated with it (Fig. 1A). The tc-genesx/gx < 1 for g1 and g6, this indicates that most drugs in the group on an average interact with the selective common tc-genes (Table 1). The tc-genesx/gx ~ 2 for g3 and g4, whereas ~ 3 for g5 and g6 suggesting that on an average g2-g6 molecules associate with more than one tc-genes (Fig. 1A).

DNA binding is the preferential activity of functional tc-genes1 (Fig. 1B) while tc-genes2 shows selective inclination towards voltage-gated calcium channels (VGCC) and metal binding activity (Fig. 1C). VGCC need calcium, a type of metal, therefore, both the MF are related^36^ and are most prevalent among tc-genes2 (Fig. 1C). In contrast to tc-genes1 and tc-genes2, acetylcholine and G-protein coupled receptor (GPCR) are the preferred actions of tc-genes3 (Fig. 1D). The GPCR’s heterodimerization and Zn (II) ions are essential for the function^37^, thus are among the significant MF associated with the tc-genes3.

Similar to tc-genes2, tc-genes4 also has a preference for VGCC and metal ion binding activity (Fig. 1E). Maximum adrenergic receptor activity is related to tc-genes5 (Fig. 1F), however, it is one of the most common MF among the tc-genes except tc-genes1. This suggests one of the possibilities is that diverse molecules can bind adrenergic receptors and the binding sites may be different. The epinephrine binding is the most preferential activity of the tc-genes6 (Fig. 1G). The sub-section indicates a molecule can be associated with many functional tc-genes’s activities and the tc-genesx is often connected with selective MF in spite of many possibilities.

### GO: CC analyses

**Figure 2:**
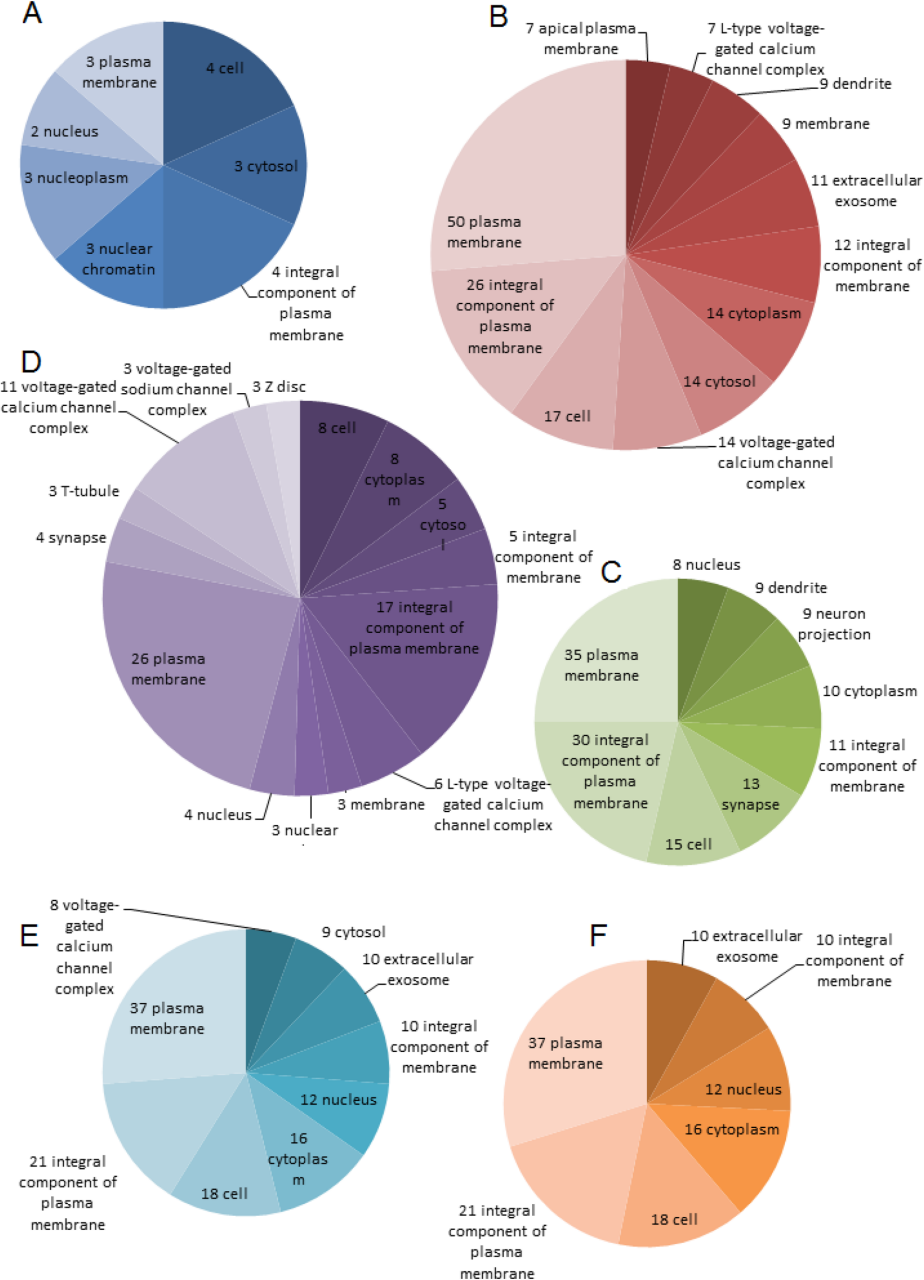
GO: CC analyses. Pie charts demonstrate the distribution of the highly occupied CC associated with (**A**) tc-genes1: dark blue, (**B**) tc-genes2: maroon, (**C**) tc-genes3: green, (**D**) tc-genes4: violet, (**E**) tc-genes5: light blue and (**F**) tc-genes6: orange, respectively. The color code for each group is as for Fig. 1. The number of instances of each CC for a group is written at the beginning.

Following the MF activities analyses, it is fascinating to investigate the CC, the location where the functional tc-genes are active. The sub-section discusses CC of the tc-gene products (proteins) of each group (Fig. 2). It is observed that plasma membrane and integral component of the plasma membrane/membrane are the common CC for all tc-genes. As plasma membrane is involved in general processes such as (i) a physical barrier to separate cytoplasm and extracellular fluids, (ii) selective permeability to certain molecules, (iii) managing endocytosis and exocytosis and (iv) facilitate cell signaling. Therefore, to find a pattern specific to tc-genesx, other CC is considered.

The preferred CC for tc-genes1 is nucleus related (Fig. 2A). This is in accordance with the MF analyses in the previous sub-section where DNA binding related activities are prevalent for tc-genes1. Most DNA in the form of chromatins is located in the nucleoplasm, a gelatinous substance inside the nucleus. Figure 2B suggests that the preferred location of action for tc-genes2 is VGCC related. Most of the VGCC complex is located on the dendrites of the neurons^38^. Exosomes are released from the cell cytoplasm by a calcium-dependent mechanism. Most of the tc-genes3 is active on the synapse, neuronal junction, a site of transmission of electrical or chemical impulse between two nerve cells (neurons) or neuron segments (dendrites) (Fig. 2C). Similar to tc-genes2, most of the functional tc-genes4 is active at VGCC complex (Fig. 2D). This suggests that the MF and CC are substantially related. In addition, the tc-genesx has a preference for selective CC and is unrandom.

### AHs: tc-genes analyses

**Figure 3:**
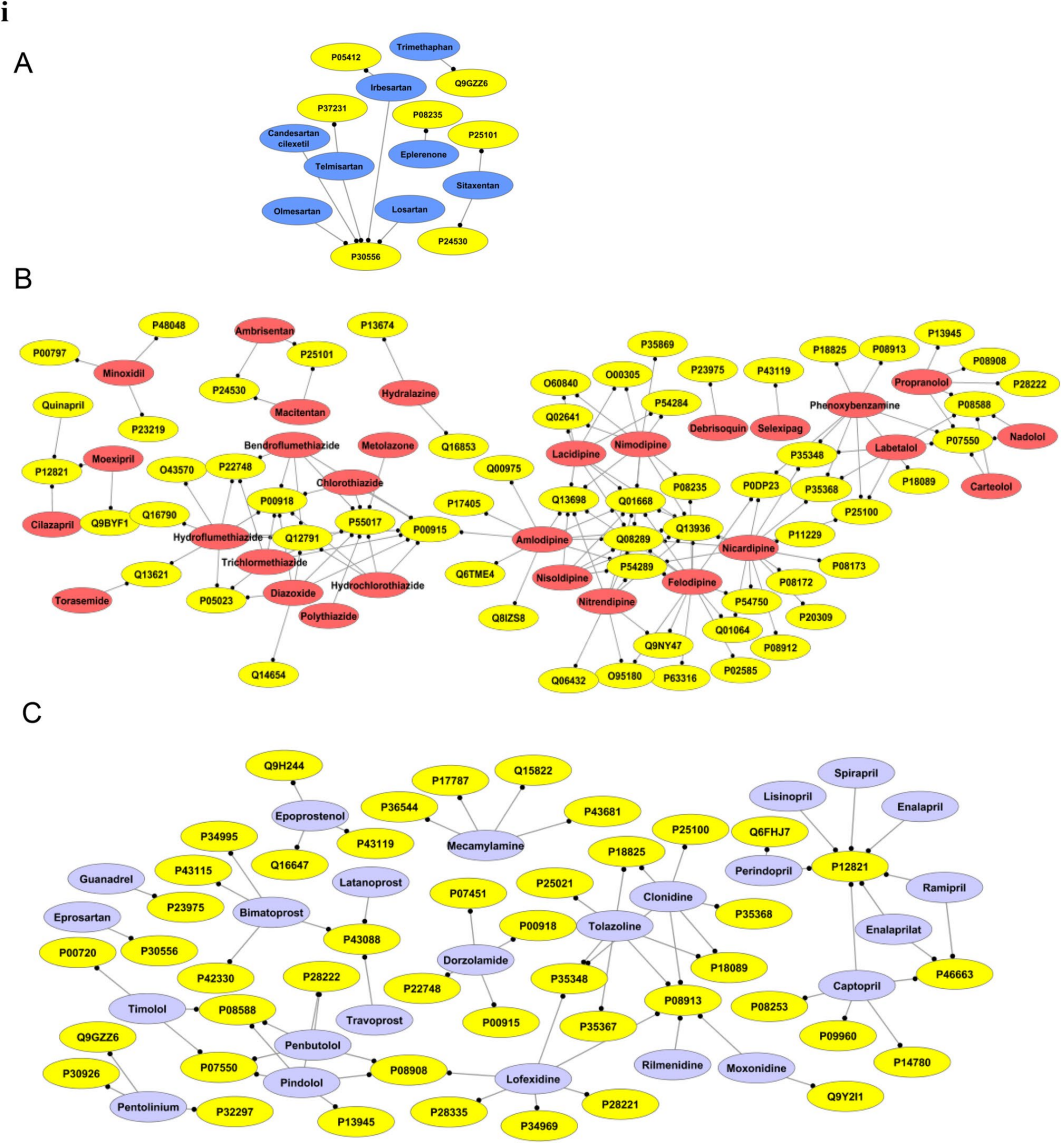

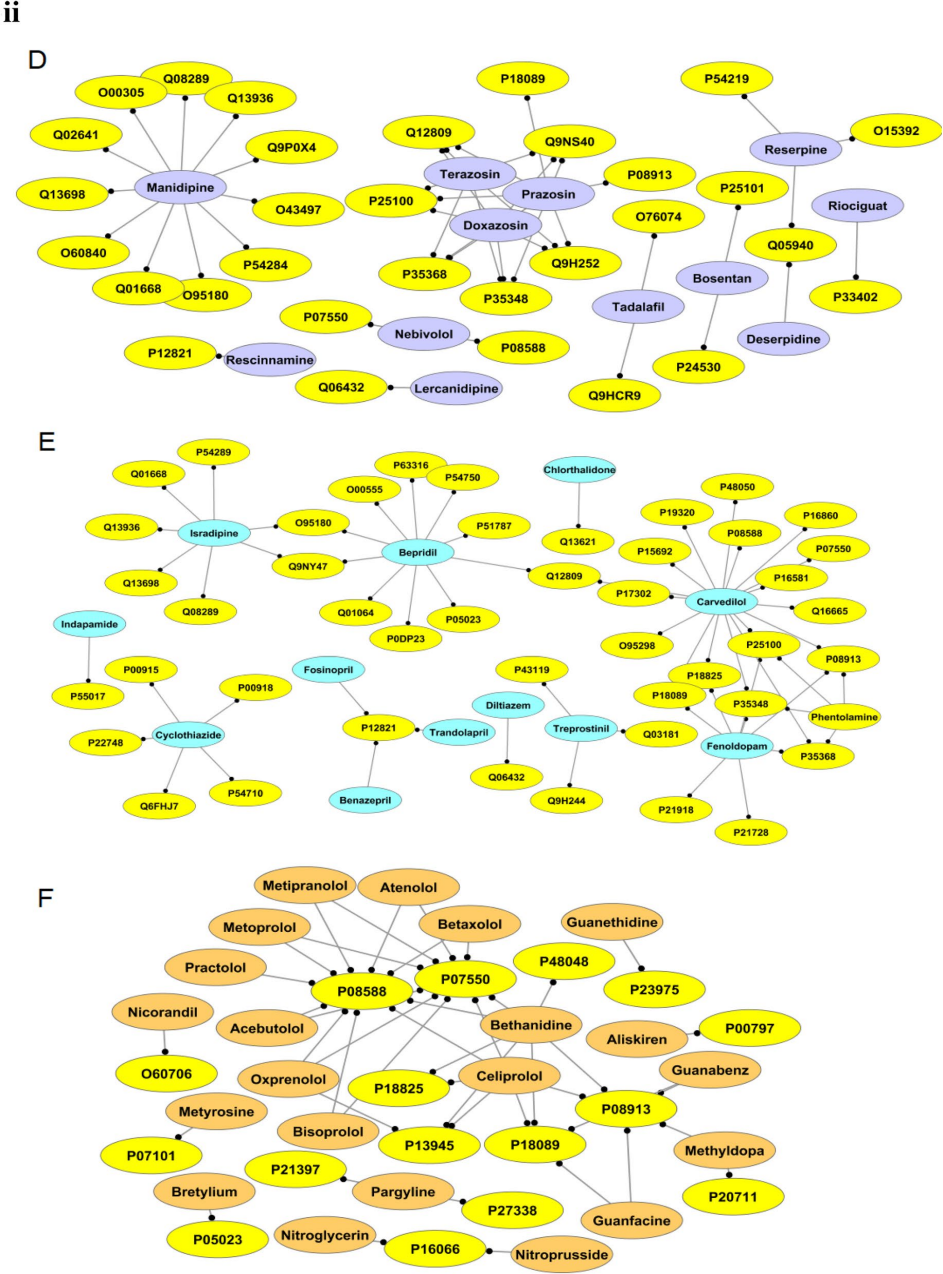
AHs: tc-genes network. **(i)** (**A**–**C**) represents the AHs: tc-genes network in a group. The medicines as nodes of A-C are coded as (**A**) g1: dark blue, (**B**) g2: maroon and (**C**) g3: violet, respectively. **(ii)** (**D**–**F**) represents the AHs: tc-genes network in a group. The medicines as nodes of D-F are coded as (**D**) g4: violet, (**E**) g5: light blue and (**F**) g6: orange, respectively. Yellow nodes code for the targets. The details of AHs and targets are listed in Table S2.

After analyzing the MF and CC related to tc-genes of each group, this sub-section investigates the connection between AHs and the functional tc-genes. Most (~ 62%) of the g1 are associated with AGTR1 that codes for type-1 angiotensin II receptor (P30556) (Fig. 3iA, Table S2). Irbesartan (DB01029), telmisartan (DB00966), olmesartan (DB00275) and losartan (DB00678) of g1 are phenyl-imidazole derivatives. They inhibit the action of angiotensin II through the renin-angiotensin system, consequently reduce the arterial blood pressure^39^. This suggests that several g1 molecules are associated with a tc-genes1, AGTR1. Most of the tc-genes1 performs transcription factor activity. In contrast to g1, only two g2 molecules (nicardipine (DB00622) and felodipine (DB01023), dihydropyridinecarboxylic acid derivatives) are associated with fifteen and thirteen tc-genes2, respectively (Fig. 3iB). Most of the common tc-genes2 codes for acetylcholine, β-adrenergic receptors and VGCC subunit. The unique targets and tc-genes associated with groups are listed in Table S3.

Angiotensin converting enzyme (P12821; ACE) is targeted by most (~ one-third) of the g3 molecules (Fig. 3iC, Table S2). These molecules are dipeptide derivatives having five and six-membered rings. Following ACE, α-2A adrenergic receptor (P08913; ADRA2A) mediates the catecholamine-induced inhibition of adenylate cyclase through G-protein’s action^40^. Imidazoline/oxazoline derivatives inhibit the activity of the protein. Manidipine (DB09238) of g4, a diphenylmethanes derivative, act as a calcium channel blocker is associated with the most (~ one-third) of the tc-genes4 involved in the common pathway of VGCC (Fig. 3iiD, Table S2).

Approximately one-third of the tc-genes3 is associated with carvedilol (DB01136), a carbazoles derivative (Fig. 3iiE). Most (~ 47%) of the tc-genes3 codes for adrenergic receptors. Bepridil (DB01244), a benzylamine derivative additionally has an association with noticeable (one-fourth) tc-genes5 (Fig. 3iiE). Approximately half of the tc-genes5 is related to VGCC and two-third is based on calcium dependent mechanism. Nearly 50% of the g6 molecules interact with β-1 adrenergic receptor (P08588; ADRB1) and most of the tc-genes6 codes for a type of adrenergic receptors. Nine of the g6 interact with β-1 adrenergic receptor (P08588; ADRB1) and β-2 adrenergic receptor (P07550; ADRB2) (Fig. 3iiF, Table S2). This suggests that only selective between AHs and tc-genes are predominant (Fig. 3).

### Common tc-genes among groups

**Figure 4:**
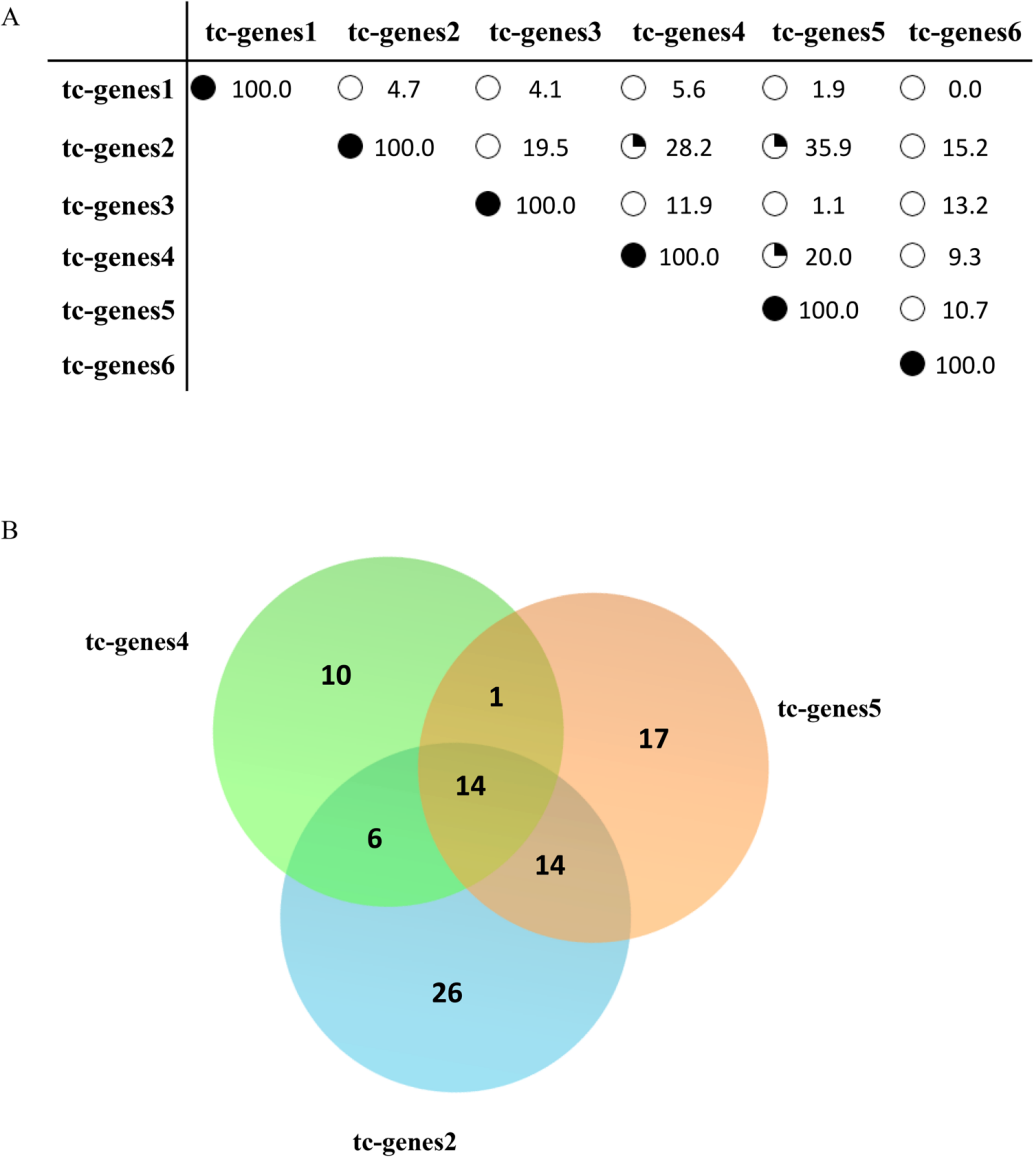
Representation of the common tc-genes. (**A**) Matrix depicts the percentage of common tc-genes. The open, one-fourth, half and full filled circle refer the approximate percentage of common molecules as of the row, for easy visualization, (**B**) Venn diagram showing common tc-genes among tc-genes2, tc-genes4 and tc-genes5.

Some of the similar MF or CC of the tc-genes can be justified if the tc-genes among the groups are common. Having common tc-genes also indicate their interaction among them in enhancing or inhibiting certain function as an anti-hypertensive. Therefore, the Cg (%) among the groups is listed in Fig. 4A in the form of a matrix. The tc-genes1 does not share a significant overlap of tc-genes among other tc-genes. The observation is in correlation with the distinct broad MF and CC the tc-genes1 possesses. Nearly one-fourth of the tc-genes2 is common with tc-genes4 and tc-genes5 (Fig. 4B, Table S4). The tc-genes4 also share approximately one-fourth of the genes with tc-genes5. Out of the common tc-genes, CAC*, ACE and ADR* are related to VGCC, angiotensin I converting enzyme and adrenergic receptors, respectively. The symbol “*” indicates anything after the character. The rest of the tc-genes share negligible overlap considering ~ 5% of error in assigning the correct group owing to the false negatives and positives in the cluster^20^. The overlapping tc-genes could be one of the reasons for similar MF or CC among tc-genes2, tc-genes4 and tc-genes5.

### Association of the tc-genes with diseases

The sub-section investigates the relationship between tc-genesx to the relevant health conditions. The details are in the “Material and methods” section.

**Figure 5:**
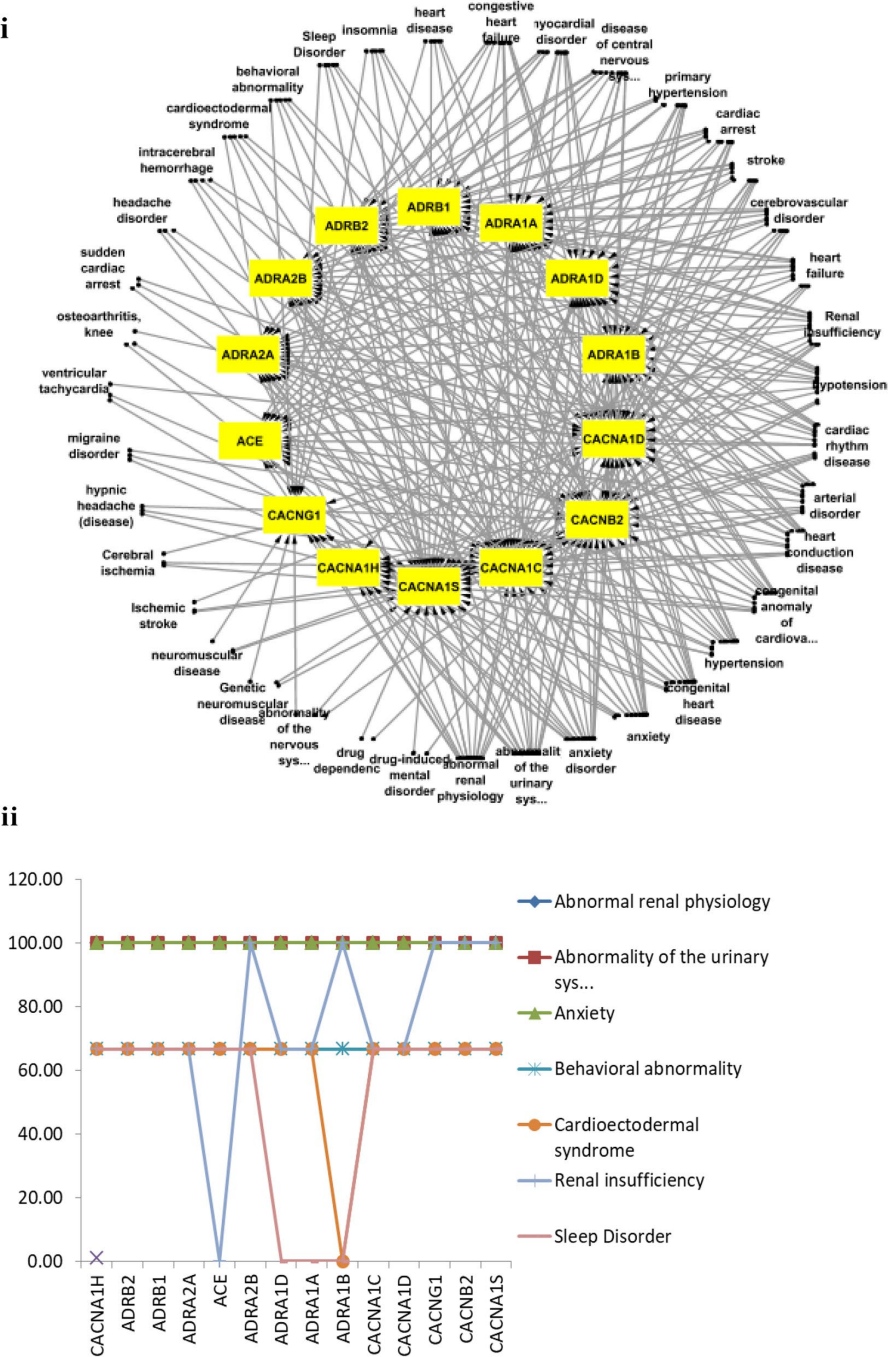
(**i**) Network of the common tc-genes and diseases. The common tc-genes and health conditions are as yellow and white nodes, respectively. The full form of the health conditions is available in Table S5. (**ii**) Common tc-genes and health conditions. The y and x-axis represents the percentage of association with the common tc-genes. The full form of the health conditions is listed in Table S5.

All the fourteen tc-genes common among tc-genes2, tc-genes4 and tc-genes5 are associated with anxiety, abnormality in renal physiology and urinary system (Fig. 5i,ii). Nearly two-thirds (~ 70%) of the tc-genes is associated with behavioral abnormality, cardioectodermal syndrome and sleeping disorders. Out of the fourteen, all except adrenergic receptors (ADRA1D, ADRA1A and ADRA1B) show a link with the sleep disorder. Regarding cardioectodermal syndrome all except ADRA1B are likely to be associated with it (Fig. 5ii). The complex network of common tc-genes is simplified in Fig. 5ii, the analyses suggest that limited tc-genes are associated with significant number of health conditions.

**Figure 6:**
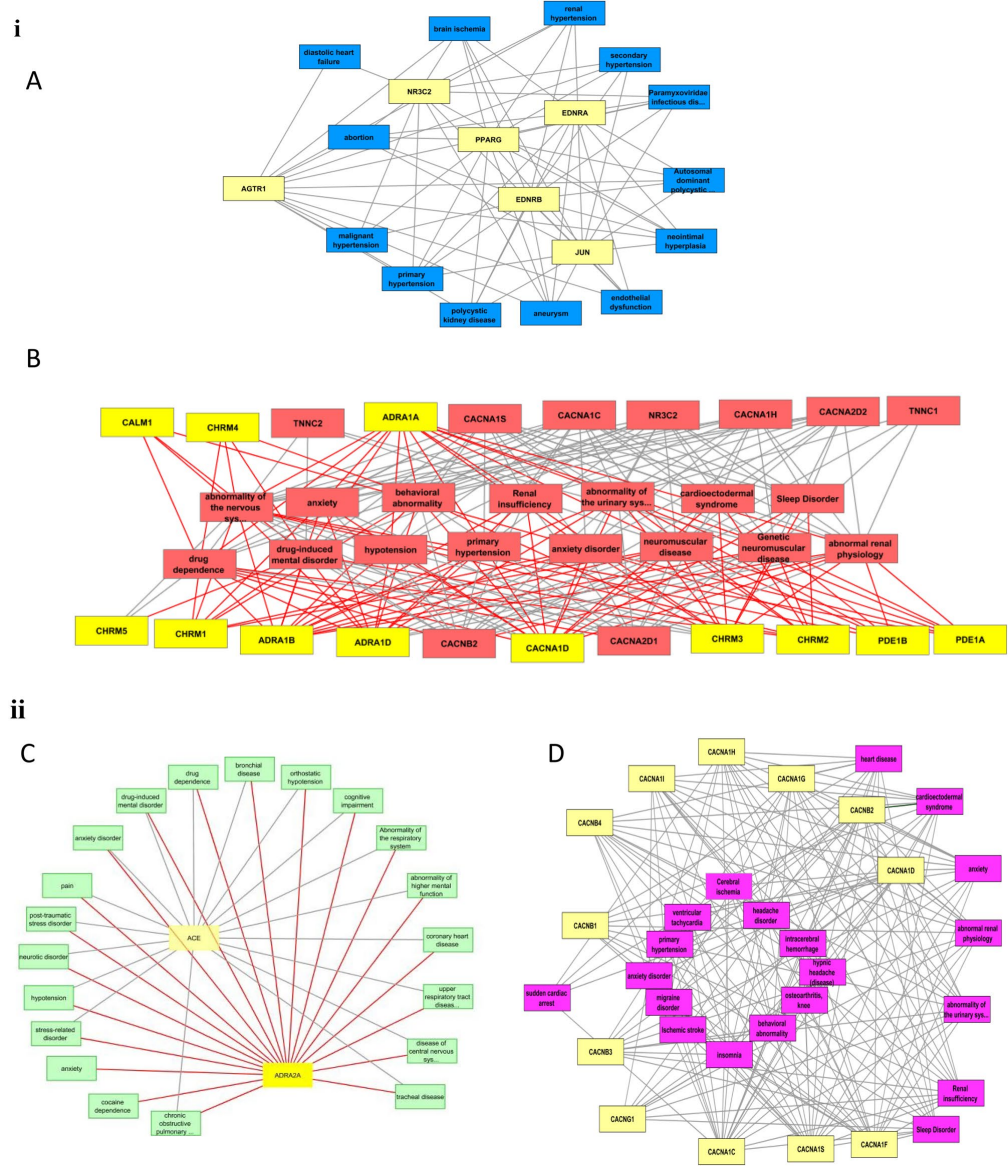

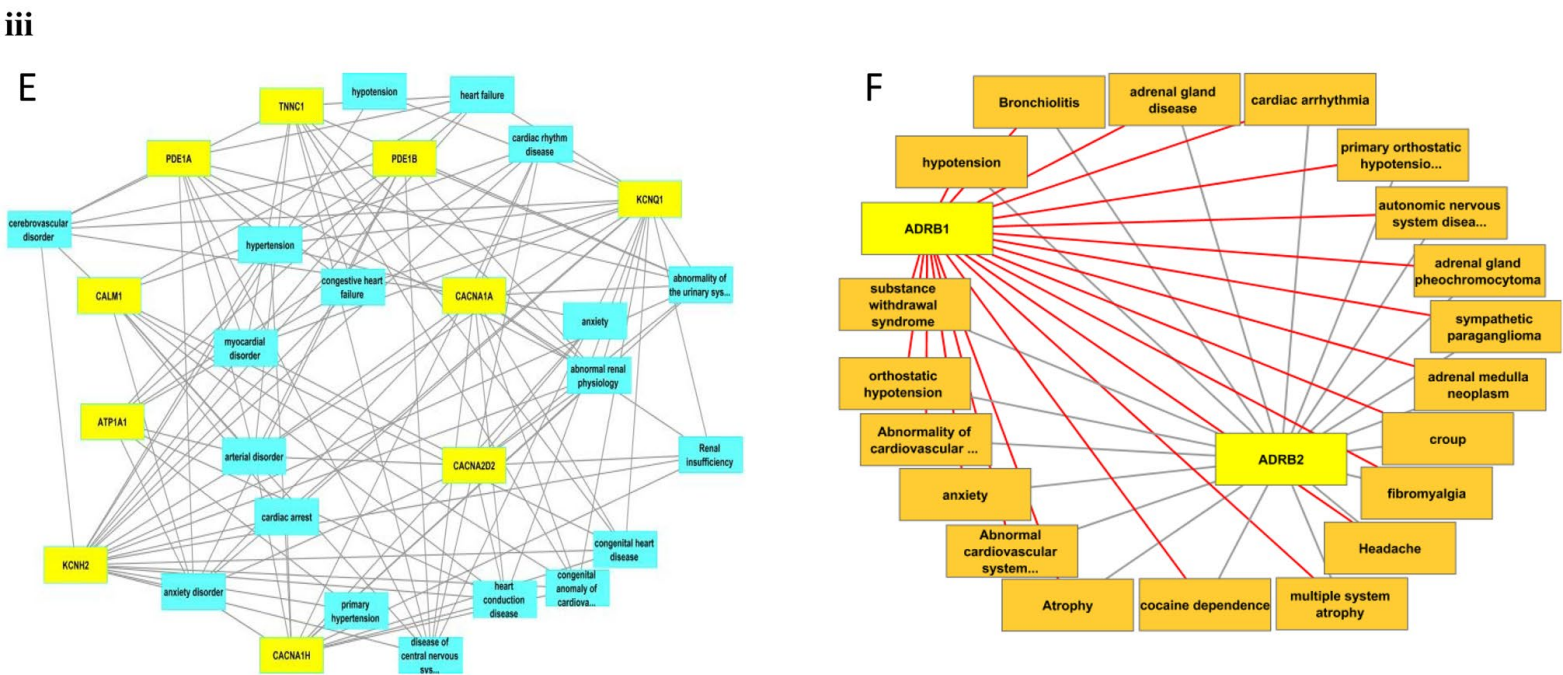
The tc-genesx: disease relationship. (**i**) Association of selective (**A**) tc-genes1 (yellow): disease (dark blue) and (**B**) tc-genes2: disease network (maroon). The tc-genes2 associated with nicardipine and felodipine is represented as yellow and maroon, respectively. (**ii**) Association of selective (**C**) tc-genes3 (yellow): disease (green) network and (**D**) tc-genes4 (yellow): disease (magenta) network. The interaction between ACE and ADRA2A are represented as grey and red edge, respectively in (**C**). (**iii**) Association of selective (**E**) tc-genes5 (yellow): disease (light blue) network and (**F**) tc-genes6 (yellow): disease (orange) network. The interaction between ADRB1 and ADRB2 are represented as red and grey edges, respectively in (**F**). The full form of the diseases is listed in Table S5.

After analyzing the health conditions associated with common tc-genes, it is interesting to find the relationship between tc-genesx to the health states. AGTR1, the most redundant among tc-genes1 is linked with all the top 20 health conditions. Aneurysm, brain ischemia, abortion, endothelial dysfunction and ischemia reperfusion injury are among the unique health conditions associated with the tc-genes1 (Fig. 6iA, Table S5). Some health conditions appear to share a link among each other too. For example, weakening of an artery wall (aneurysm) or pathological state of the inner lining of the blood vessels (endothelial dysfunction) may disrupt the flow of blood to the organs, namely, the brain (brain ischemia) or uterus, therefore can lead to stroke or abortion, respectively. Surprisingly, most (~ 95%) of the tc-genes1 is associated with all the health conditions except diastolic heart failure condition. The later is likely to be associated with only two tc-genes1, ADTR1 and NR3C2 (Fig. 6iA). However, one of the reasons for diastolic heart failure can be hardening of the arteries (aneurysm)^41^. This suggests some of the health conditions can be associated with other health conditions. Also, a few of the health conditions have a unique association to tc-genes1.

Most of the common tc-genes2 is associated with integral membrane proteins, viz., acetylcholine receptor, β-adrenergic receptors and VGCC. Apart from the binding of acetylcholine, acetylcholine receptors respond to other molecules as well, for example nicotine^42^. Therefore, nicotine dependence, drug dependence, substance related and drug induced mental disorders are among most relevant health conditions associated with tc-genes2 (Fig. 6iB). Adrenergic receptors are a type of GPCR that targets catecholamines like norepinephrine and epinephrine, which are part of sympathetic nervous system. Adrenal medulla locates on the kidneys and regulates the secretion of the molecules at the time of anxiety or stress. Epinephrine and norepinephrine affect heart, lungs, muscles and blood vessels, which in turn have related health conditions. VGCC are group of voltage regulated ion channels with permeability to calcium ions. GWAS analysis supports CACNA1C’s participation in bipolar disorder, a behavioral disorder related to abnormal depression and elevated moods^43^. Anxiety and substance related disorders are commonly associated with bipolar disorders^44^. This suggests some health conditions are connected.

ACE and ADRA2A are among tc-genes3 that code for angiotensin converting enzyme and α-2A adrenergic receptor, respectively. Angiotensin converting enzyme participates in the renin-angiotensin system to convert angiotensin I to angiotensin II. The latter constricts the blood vessels and stimulates the production of aldosterone that regulates the fluid balance by the kidneys. In addition, angiotensin II aids in kidney development by affecting growth factors. Mutations in ACE can cause severe kidney related disorders, stroke and heart failure^45–47^. A recent study also supports that the renin-angiotensin system performs a critical role in causing heart failure^48^ (Fig. 6iiC). This indicates that similar to tc-genes1 and tc-genes2, a few of the tc-genes3 are also associated with unique health conditions.

Although most tc-genes4 participates in a common pathway, that is, voltage gated channels. However, the health condition may or may not be associated with all twelve tc-genes. Heart related conditions involve a sudden cardiac arrest, ventricular tachycardia, cardioelectodermal syndrome and heart disease. Sudden cardiac arrest is associated with only five genes, CACNA1H, CACNA1G, CACNB2, CACNA1D and CACNA1C. Although ventricular tachycardia remain a heart related ailment, but is linked with common tc-genes (CACNA1C and CACNA1D). Cardioectodermal syndrome and heart disease are linked to all twelve tc-genes4 (Fig. 6iiD). Unlike heart related ailments, kidney related health conditions such as abnormality of the urinary system, renal insufficiency and abnormal renal physiology are associated with all the tc-genes. In contrast to kidney related conditions, brain related disorders such as cerebral ischemia, intracerebral hemorrhage and ischemic stroke are associated with common tc-genes (CACNA1C, CACNA1D, CACNA1S, and CACNA1F). Intracerebral hemorrhage is associated with an additional tc-gene, CACNB2. Out of a few tc-genes, CACNA1C and CACNA1D are the common genes affecting heart, brain and kidney followed by CSCNA1S. This suggests that one form of health condition is related to other health conditions through common tc-genes.

Most tc-genes5 instructs to form voltage gated channels and adrenoreceptors. KCNH2 and KCNQ1 are associated with all the top relevant health conditions associated with tc-genes5 (Fig. 6iiiE, Table S5). It codes for the voltage gated ion channels that modulate the neuronal excitability and neurotransmitter release, thus participates in the generation of action potential. The role of VGCC has been discussed in previous sub-section describing its relation to heart related disorders. As discussed in the previous sub-sections, ADRB1 and ADRB2 are the major interacting tc-genes among tc-genes6 that code for β-1 adrenergic receptor and β-2 adrenergic receptor, respectively. A disorder related to muscular pain, stiffness and tenderness (fibromyalgia), partial or complete waste away of certain parts (atrophy), infection of the upper airway (croup) and adrenal related health conditions (adrenal gland pheochromocytoma, adrenal medulla neoplasm and adrenal gland disease) are among the most relevant and are unique to tc-genes6 (Fig. 6iiiF). Adrenal gland constitutes adrenergic and non-adrenergic chromaffin cells. All the tc-genes6 is associated with adrenal gland disease. This suggests that a few of the health conditions are specific to a tc-genesx whereas the rest are common among tc-genes.

### Association between tc-genes and organ related health conditions

**Figure 7:**
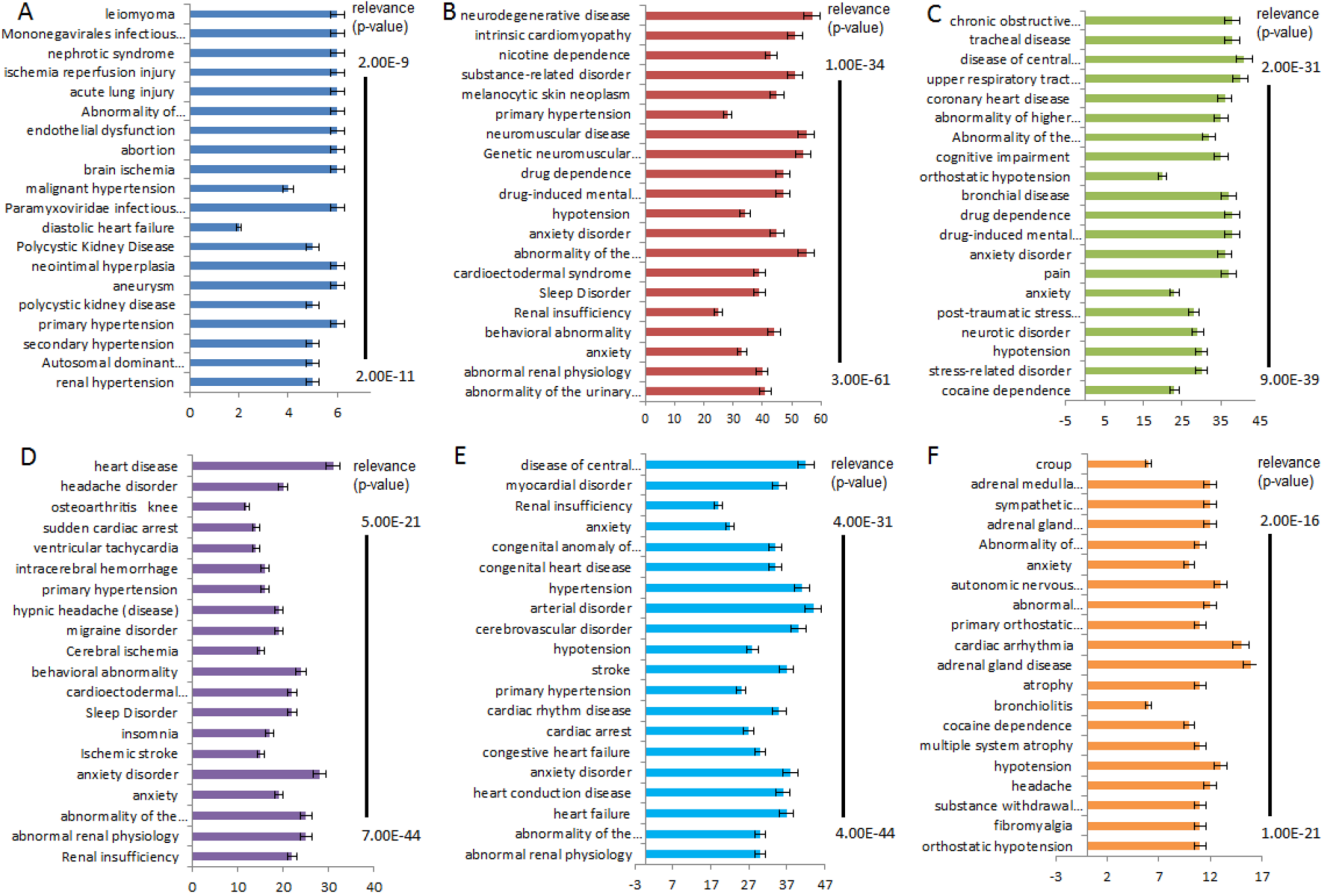
Distribution of the tc-genes associated with the health conditions. (**A**–**F**) The bar graph represents the number of tc-genes (x-axis) associated with the top 20 health conditions (y-axis). The color code of each group is the same as for Fig. 1. The full form of abbreviated health conditions is listed in the supplementary information, Table S5.

Renal related ailment tops the chart, showing maximum association to all except tc-genes3 and tc-genes6 (Fig. 7). All except tc-genes6 affect kidney, heart and brain related health conditions. Unlike the rest, tc-genes6 shows an association with the adrenal glands (Table S5). The tc-genes2 and tc-genes3 list the maximum number of abnormality related to the nervous system. Most tc-genes4 is related to a heart ailment. Arterial disorder, followed by the central nervous system related health condition is associated with most tc-genes5. Although all AHs are associated primarily with kidney, heart and brain, but the specific type of disorder is typically restricted to each group (Fig. 7). The analyses of this sub-section suggest that more than one tc-gene within a group is frequently associated with similar health conditions.

### Experimentally validated combinations of AHs

Following analyses of the association between tc-genes and health conditions, the sub-section investigates the known experimentally validated combinations of AHs reported in Cheng et al.^8^ to identify the trend in successful drug–drug combinations for treating the health condition.

**Figure 8:**
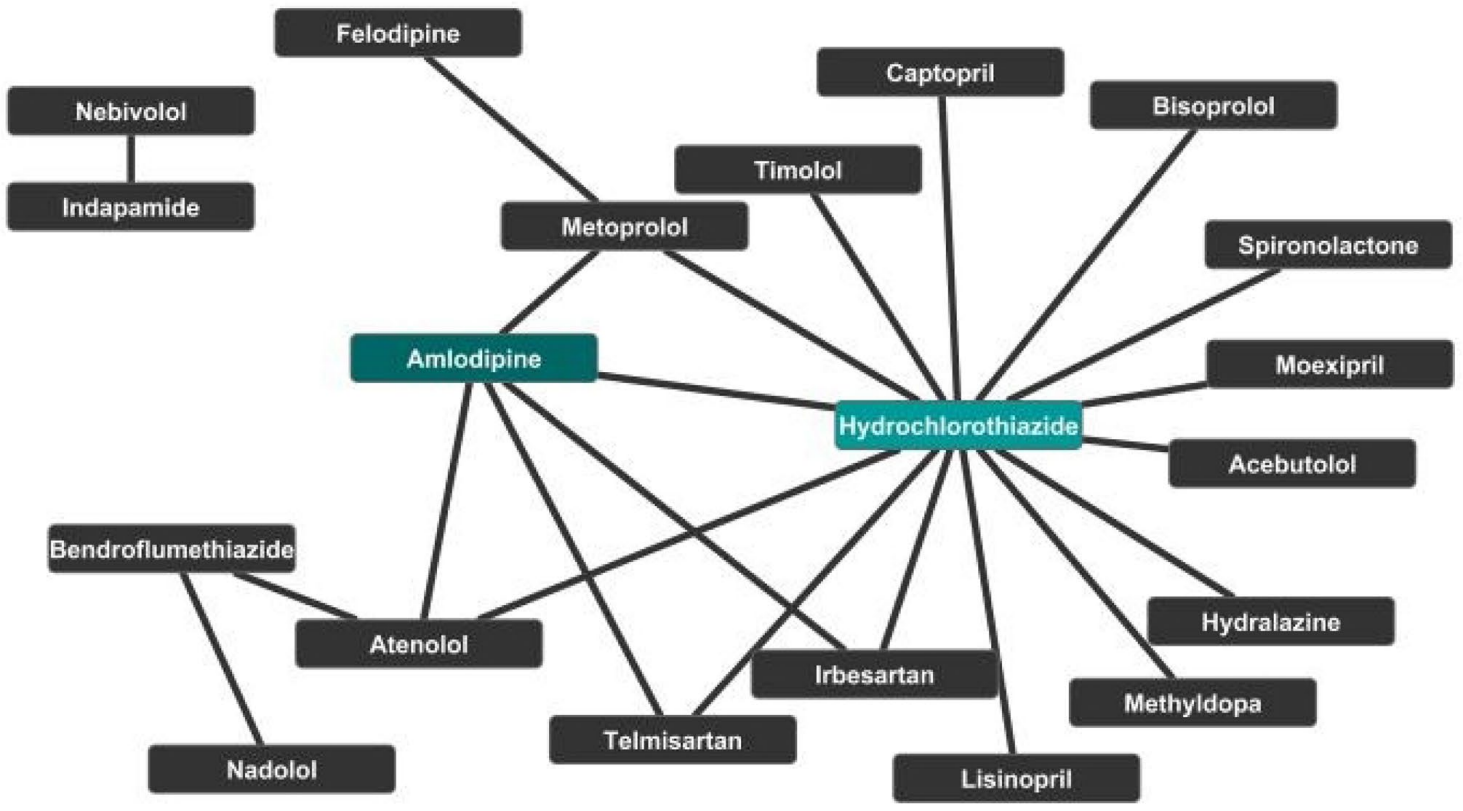
Network of selective experimentally validated drug–drug combinations for treating hypertension. The AHs are represented as black nodes except amlodipine and hydrochlorothiazide, which are in dark green background.

**Figure 9:**
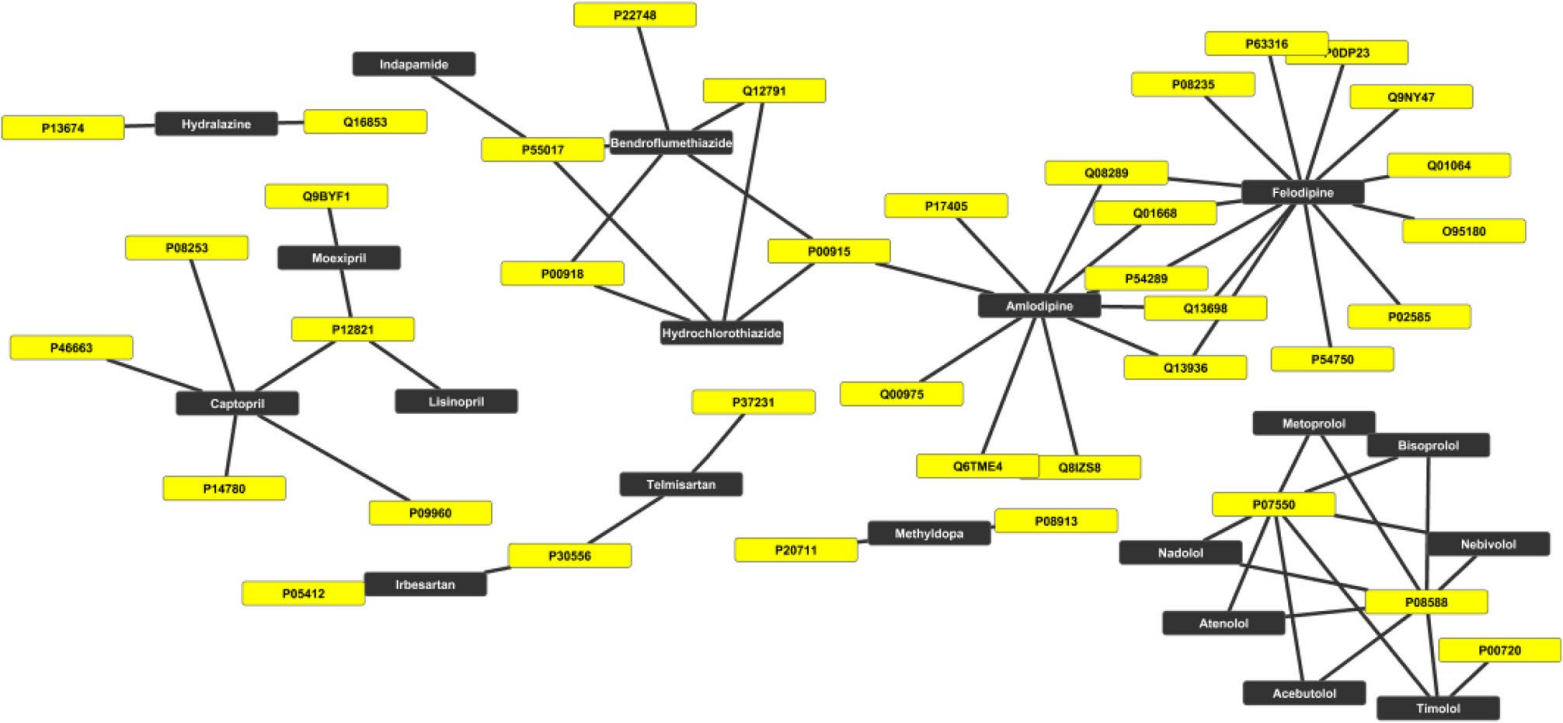
Network of AHs prescribed in drug–drug combinations and targets. The nodes of the targets and AHs are in the yellow and black background, respectively.

The twenty-one experimentally validated drug–drug combinations for treating hypertension involve nineteen unique AHs (Table S6). Out of these, more than two-thirds belong to g2. Hydrochlorothiazide and amlodipine provide the maximum number of approved combinations interacting with nearly two-third (~ 61%) and one-fourth (~ 19%) of them, respectively (Fig. 8). Hydrochlorothiazide and amlodipine have nearly one-fourth (~ 23%) of the same combination partners including each other (Fig. 8). Despite having high partners (fifteen), hydrochlorothiazide shows interaction with only four targets (Fig. 7). On the other hand, amlodipine interacts with more targets (nine) than approved combinations (four). Although felodipine has only one reported drug–drug combination but binds with most targets among AHs involved in clinically approved combinations (Fig. 9). Approximately one-third (~ 36%) of the AHs involved as combinations interact with a β-2 adrenergic receptor (P07550; ADRB1) and β-1 adrenergic receptor (P08588; ADRB2). Both carry out agonist and the antagonist type of activity. ADRB1 and ADRB2 occupy separate sites for the highest level of expression; for ADRB1 and ADRB2, it is squamous epithelium and placenta, respectively. It is crucial to note we observed that successfully clinically validated AHs combinations often interact with various targets (Fig. 8).

Nearly half of the pairwise combinations are between g2 and g6 molecules. Except for g2 molecules, no other groups have clinically validated combinations within them. Nearly one-fourth (~ 23%) of the combinations are within g2 molecules. The g2 molecules deliver the maximum number of combinations. Analyses of the experimentally validated combinations suggest the following trend; g2 > g6 > g1 > g3 = g4 = g5 in producing successful drug combinations (Table S6). This suggests the preference of AHs in obtaining drug–drug combinations.

In the recent studies, Guo et al.^49^ incorporated network based genome-wide gene prediction methods while Li et al.^50^ assigned the topology and agent scores to identify potential drug–drug combinations. In contrast, our study identifies the pattern associated with the successful combinations treating hypertension.

The prior clustering of the AHs followed by a network based approach of AHs: tc-genes: diseases aids in deciphering the relationship between hypertension to other health conditions. The correct clustering of the molecules is salient (ignoring ~ 5% of error) and can relate the structural patterns to its function. The reverse tracing the action of AHs identifies the tc-genes which in turn are associated with health conditions.

## Conclusion

The paper reports the interplay among AHs: tc-genes: diseases through network based approach. Recent experimental studies augment our findings so far. Overall, the study suggests that tc-genes associated with AHs groups have a preference in MF and CC activities. There are many AHs interacting selective common targets as well as a few AHs are interacting with many targets. Most AHs interacting with distinct targets are involved in the same pathway. The top health conditions associated with tc-genes are mainly renal, heart, brain or lung related conditions. There are trends for successful drug–drug combinations and these pairs bind separate targets.

## Supplementary information

Supplementary Information.
