# Supplementary material for: Network-based approach highlighting interplay among anti-hypertensives: target coding-genes: diseases
Source: Sci Rep. 2020 Nov 19;10:20152. doi: 10.1038/s41598-020-76605-1 (PMC7677320; doi:10.1038/s41598-020-76605-1)
Supplement: Supplementary file 1 — Supplementary Information. [file 41598_2020_76605_MOESM1_ESM.doc]

**Supplementary Information**

**Network-based Approach Highlighting Interplay among Anti-hypertensives: Target-coding Genes: Diseases**

**Supplementary Tables**

**Table S1.** List of the AHs considered for the study. AHs are clustered into six groups (k=6) as mentioned in Sharma, 2020 [23].

**Table S2.** Details of the AHs and targets associated to the groups.

**Table S3.** List of the unique targets and tc-genes IDs associated with groups.

**Table S4.** List of the common tc-genes among tc-genes2, tc-genes4 and tc-genes5.

**Table S5.** Top 20 health conditions associated with tc-genesx (p < 2.0E-9).

**Table S6.** List of gold-standard pairwise combinations of the AHs.

**Table S1.** List of the AHs considered for the study. AHs are clustered into six groups (k=6) as mentioned in Sharma, 2020 [23].

| **S. No.** | **DB_ID** | **Name** | **k=6** |
| --- | --- | --- | --- |
| 1 | DB00177 | Valsartan | 5 |
| 2 | DB00178 | Ramipril | 3 |
| 3 | DB00187 | Esmolol | 6 |
| 4 | DB00195 | Betaxolol | 6 |
| 5 | DB00206 | Reserpine | 4 |
| 6 | DB00214 | Torasemide | 2 |
| 7 | DB00217 | Bethanidine | 6 |
| 8 | DB00226 | Guanadrel | 3 |
| 9 | DB00264 | Metoprolol | 6 |
| 10 | DB00270 | Isradipine | 5 |
| 11 | DB00275 | Olmesartan | 1 |
| 12 | DB00287 | Travoprost | 3 |
| 13 | DB00310 | Chlorthalidone | 5 |
| 14 | DB00325 | Nitroprusside | 6 |
| 15 | DB00335 | Atenolol | 6 |
| 16 | DB00343 | Diltiazem | 5 |
| 17 | DB00350 | Minoxidil | 2 |
| 18 | DB00373 | Timolol | 3 |
| 19 | DB00374 | Treprostinil | 5 |
| 20 | DB00381 | Amlodipine | 2 |
| 21 | DB00393 | Nimodipine | 2 |
| 22 | DB00401 | Nisoldipine | 2 |
| 23 | DB00436 | Bendroflumethiazide | 2 |
| 24 | DB00457 | Prazosin | 4 |
| 25 | DB00492 | Fosinopril | 5 |
| 26 | DB00519 | Trandolapril | 5 |
| 27 | DB00521 | Carteolol | 2 |
| 28 | DB00524 | Metolazone | 2 |
| 29 | DB00528 | Lercanidipine | 4 |
| 30 | DB00542 | Benazepril | 5 |
| 31 | DB00559 | Bosentan | 4 |
| 32 | DB00571 | Propranolol | 2 |
| 33 | DB00575 | Clonidine | 3 |
| 34 | DB00584 | Enalapril | 3 |
| 35 | DB00590 | Doxazosin | 4 |
| 36 | DB00598 | Labetalol | 2 |
| 37 | DB00606 | Cyclothiazide | 5 |
| 38 | DB00612 | Bisoprolol | 6 |
| 39 | DB00622 | Nicardipine | 2 |
| 40 | DB00629 | Guanabenz | 6 |
| 41 | DB00654 | Latanoprost | 3 |
| 42 | DB00657 | Mecamylamine | 3 |
| 43 | DB00678 | Losartan | 1 |
| 44 | DB00691 | Moexipril | 2 |
| 45 | DB00692 | Phentolamine | 5 |
| 46 | DB00700 | Eplerenone | 1 |
| 47 | DB00722 | Lisinopril | 3 |
| 48 | DB00727 | Nitroglycerin | 6 |
| 49 | DB00765 | Metyrosine | 6 |
| 50 | DB00774 | Hydroflumethiazide | 2 |
| 51 | DB00790 | Perindopril | 3 |
| 52 | DB00796 | Candesartan cilexetil | 1 |
| 53 | DB00797 | Tolazoline | 3 |
| 54 | DB00800 | Fenoldopam | 5 |
| 55 | DB00808 | Indapamide | 5 |
| 56 | DB00820 | Tadalafil | 4 |
| 57 | DB010869 | Dorzolamide | 3 |
| 58 | DB00876 | Eprosartan | 3 |
| 59 | DB00880 | Chlorothiazide | 2 |
| 60 | DB00881 | Quinapril | 2 |
| 61 | DB00905 | Bimatoprost | 3 |
| 62 | DB00925 | Phenoxybenzamine | 2 |
| 63 | DB00960 | Pindolol | 3 |
| 64 | DB00966 | Telmisartan | 1 |
| 65 | DB00968 | Methyldopa | 6 |
| 66 | DB00999 | Hydrochlorothiazide | 2 |
| 67 | DB01018 | Guanfacine | 6 |
| 68 | DB01021 | Trichlormethiazide | 2 |
| 69 | DB01023 | Felodipine | 2 |
| 70 | DB01029 | Irbesartan | 1 |
| 71 | DB01054 | Nitrendipine | 2 |
| 72 | DB01089 | Deserpidine | 4 |
| 73 | DB01090 | Pentolinium | 3 |
| 74 | DB01116 | Trimethaphan | 1 |
| 75 | DB01119 | Diazoxide | 2 |
| 76 | DB01136 | Carvedilol | 5 |
| 77 | DB01158 | Bretylium | 6 |
| 78 | DB01162 | Terazosin | 4 |
| 79 | DB01170 | Guanethidine | 6 |
| 80 | DB01180 | Rescinnamine | 4 |
| 81 | DB01193 | Acebutolol | 6 |
| 82 | DB01197 | Captopril | 3 |
| 83 | DB01203 | Nadolol | 2 |
| 84 | DB01214 | Metipranolol | 6 |
| 85 | DB01240 | Epoprostenol | 3 |
| 86 | DB01244 | Bepridil | 5 |
| 87 | DB01275 | Hydralazine | 2 |
| 88 | DB01297 | Practolol | 6 |
| 89 | DB01324 | Polythiazide | 2 |
| 90 | DB01340 | Cilazapril | 2 |
| 91 | DB01348 | Spirapril | 3 |
| 92 | DB01359 | Penbutolol | 3 |
| 93 | DB01580 | Oxprenolol | 6 |
| 94 | DB01626 | Pargyline | 6 |
| 95 | DB04840 | Debrisoquin | 2 |
| 96 | DB04846 | Celiprolol | 6 |
| 97 | DB04861 | Nebivolol | 4 |
| 98 | DB04948 | Lofexidine | 3 |
| 99 | DB06268 | Sitaxentan | 1 |
| 100 | DB06403 | Ambrisentan | 2 |
| 101 | DB06762 | Pinacidil | 6 |
| 102 | DB06826 | Unoprostone | 6 |
| 103 | DB08931 | Riociguat | 4 |
| 104 | DB08932 | Macitentan | 2 |
| 105 | DB09026 | Aliskiren | 6 |
| 106 | DB09220 | Nicorandil | 6 |
| 107 | DB09236 | Lacidipine | 2 |
| 108 | DB09238 | Manidipine | 4 |
| 109 | DB09242 | Moxonidine | 3 |
| 110 | DB09477 | Enalaprilat | 3 |
| 111 | DB11362 | Selexipag | 2 |
| 112 | DB11738 | Rilmenidine | 3 |
| 113 | DB12945 | Dihydralazine | 2 |
| 114 | DB13532 | Cyclopenthiazide | 2 |

**Table S2.** The details of the AHs and targets associated to the groups.

| **g1** | | | |
| --- | --- | --- | --- |
| **DrugBank_ID** | **Name** | **UniProt_ID** | **Target Name** |
| DB00275 | Olmesartan | P30556 | Type-1 angiotensin II receptor |
| DB00678 | Losartan | P30556 | Type-1 angiotensin II receptor |
| DB00700 | Eplerenone | P08235 | Mineralocorticoid receptor |
| DB00796 | Candesartan cilexetil | P30556 | Type-1 angiotensin II receptor |
| DB00966 | Telmisartan | P30556 | Type-1 angiotensin II receptor |
| DB00966 | Telmisartan | P37231 | Peroxisome proliferator-activated receptor gamma |
| DB01029 | Irbesartan | P30556 | Type-1 angiotensin II receptor |
| DB01029 | Irbesartan | P05412 | Transcription factor AP-1 |
| DB01116 | Trimethaphan | Q9GZZ6 | Neuronal acetylcholine receptor subunit alpha-10 |
| DB06268 | Sitaxentan | P25101 | Endothelin-1 receptor |
| DB06268 | Sitaxentan | P24530 | Endothelin B receptor |

| **g2** | | | |
| --- | --- | --- | --- |
| **DrugBank_ID** | **Name** | **UniProt_ID** | **Target Name** |
| DB00214 | Torasemide | Q13621 | Solute carrier family 12 member 1 |
| DB00350 | Minoxidil | P48048 | ATP-sensitive inward rectifier potassium channel 1 |
| DB00350 | Minoxidil | P23219 | Prostaglandin G/H synthase 1 |
| DB00350 | Minoxidil | P00797 | Renin |
| DB00381 | Amlodipine | Q13936 | Voltage-dependent L-type calcium channel subunit alpha-1C |
| DB00381 | Amlodipine | P54289 | Voltage-dependent calcium channel subunit alpha-2/delta-1 |
| DB00381 | Amlodipine | Q08289 | Voltage-dependent L-type calcium channel subunit beta-2 |
| DB00381 | Amlodipine | Q01668 | Voltage-dependent L-type calcium channel subunit alpha-1D |
| DB00381 | Amlodipine | Q13698 | Voltage-dependent L-type calcium channel subunit alpha-1S |
| DB00381 | Amlodipine | Q00975 | Voltage-dependent N-type calcium channel subunit alpha-1B |
| DB00381 | Amlodipine | Q8IZS8 | Voltage-dependent calcium channel subunit alpha-2/delta-3 |
| DB00381 | Amlodipine | Q6TME4 | Voltage-dependent L-type calcium channel subunit beta-1 |
| DB00381 | Amlodipine | P00915 | Carbonic anhydrase 1 |
| DB00381 | Amlodipine | P17405 | Sphingomyelin phosphodiesterase |
| DB00393 | Nimodipine | Q13936 | Voltage-dependent L-type calcium channel subunit alpha-1C |
| DB00393 | Nimodipine | Q01668 | Voltage-dependent L-type calcium channel subunit alpha-1D |
| DB00393 | Nimodipine | O60840 | Voltage-dependent L-type calcium channel subunit alpha-1F |
| DB00393 | Nimodipine | Q13698 | Voltage-dependent L-type calcium channel subunit alpha-1S |
| DB00393 | Nimodipine | Q02641 | Voltage-dependent L-type calcium channel subunit beta-1 |
| DB00393 | Nimodipine | Q08289 | Voltage-dependent L-type calcium channel subunit beta-2 |
| DB00393 | Nimodipine | P54284 | Voltage-dependent L-type calcium channel subunit beta-3 |
| DB00393 | Nimodipine | O00305 | Voltage-dependent L-type calcium channel subunit beta-4 |
| DB00393 | Nimodipine | P08235 | Mineralocorticoid receptor |
| DB00393 | Nimodipine | P35869 | Aryl hydrocarbon receptor |
| DB00401 | Nisoldipine | Q13936 | Voltage-dependent L-type calcium channel subunit alpha-1C |
| DB00401 | Nisoldipine | P54289 | Voltage-dependent calcium channel subunit alpha-2/delta-1 |
| DB00401 | Nisoldipine | Q08289 | Voltage-dependent L-type calcium channel subunit beta-2 |
| DB00401 | Nisoldipine | Q01668 | Voltage-dependent L-type calcium channel subunit alpha-1D |
| DB00401 | Nisoldipine | Q13698 | Voltage-dependent L-type calcium channel subunit alpha-1S |
| DB00436 | Bendroflumethiazide | P55017 | Solute carrier family 12 member 3 |
| DB00436 | Bendroflumethiazide | Q12791 | Calcium-activated potassium channel subunit alpha-1 |
| DB00436 | Bendroflumethiazide | P00915 | Carbonic anhydrase 1 |
| DB00436 | Bendroflumethiazide | P00918 | Carbonic anhydrase 2 |
| DB00436 | Bendroflumethiazide | P22748 | Carbonic anhydrase 4 |
| DB00521 | Carteolol | P07550 | Beta-2 adrenergic receptor |
| DB00521 | Carteolol | P08588 | Beta-1 adrenergic receptor |
| DB00524 | Metolazone | P55017 | Solute carrier family 12 member 3 |
| DB00571 | Propranolol | P08588 | Beta-1 adrenergic receptor |
| DB00571 | Propranolol | P07550 | Beta-2 adrenergic receptor |
| DB00571 | Propranolol | P13945 | Beta-3 adrenergic receptor |
| DB00571 | Propranolol | P08908 | 5-hydroxytryptamine receptor 1A |
| DB00571 | Propranolol | P28222 | 5-hydroxytryptamine receptor 1B |
| DB00598 | Labetalol | P08588 | Beta-1 adrenergic receptor |
| DB00598 | Labetalol | P07550 | Beta-2 adrenergic receptor |
| DB00598 | Labetalol | P35348 | Alpha-1A adrenergic receptor |
| DB00598 | Labetalol | P35368 | Alpha-1B adrenergic receptor |
| DB00598 | Labetalol | P25100 | Alpha-1D adrenergic receptor |
| DB00622 | Nicardipine | Q13936 | Voltage-dependent L-type calcium channel subunit alpha-1C |
| DB00622 | Nicardipine | Q08289 | Voltage-dependent L-type calcium channel subunit beta-2 |
| DB00622 | Nicardipine | P54289 | Voltage-dependent calcium channel subunit alpha-2/delta-1 |
| DB00622 | Nicardipine | Q01668 | Voltage-dependent L-type calcium channel subunit alpha-1D |
| DB00622 | Nicardipine | P54750 | Calcium/calmodulin-dependent 3' |
| DB00622 | Nicardipine | Q01064 | Calcium/calmodulin-dependent 3' |
| DB00622 | Nicardipine | P35348 | Alpha-1A adrenergic receptor |
| DB00622 | Nicardipine | P35368 | Alpha-1B adrenergic receptor |
| DB00622 | Nicardipine | P25100 | Alpha-1D adrenergic receptor |
| DB00622 | Nicardipine | P11229 | Muscarinic acetylcholine receptor M1 |
| DB00622 | Nicardipine | P08172 | Muscarinic acetylcholine receptor M2 |
| DB00622 | Nicardipine | P20309 | Muscarinic acetylcholine receptor M3 |
| DB00622 | Nicardipine | P08173 | Muscarinic acetylcholine receptor M4 |
| DB00622 | Nicardipine | P08912 | Muscarinic acetylcholine receptor M5 |
| DB00622 | Nicardipine | P0DP23 | Calmodulin |
| DB00691 | Moexipril | P12821 | Angiotensin-converting enzyme |
| DB00691 | Moexipril | Q9BYF1 | Angiotensin-converting enzyme 2 |
| DB00774 | Hydroflumethiazide | Q13621 | Solute carrier family 12 member 1 |
| DB00774 | Hydroflumethiazide | P00915 | Carbonic anhydrase 1 |
| DB00774 | Hydroflumethiazide | P00918 | Carbonic anhydrase 2 |
| DB00774 | Hydroflumethiazide | P22748 | Carbonic anhydrase 4 |
| DB00774 | Hydroflumethiazide | Q16790 | Carbonic anhydrase 9 |
| DB00774 | Hydroflumethiazide | O43570 | Carbonic anhydrase 12 |
| DB00774 | Hydroflumethiazide | P05023 | Sodium/potassium-transporting ATPase subunit alpha-1 |
| DB00774 | Hydroflumethiazide | Q12791 | Calcium-activated potassium channel subunit alpha-1 |
| DB00880 | Chlorothiazide | P55017 | Solute carrier family 12 member 3 |
| DB00880 | Chlorothiazide | P00915 | Carbonic anhydrase 1 |
| DB00880 | Chlorothiazide | P00918 | Carbonic anhydrase 2 |
| DB00881 | Quinapril | P12821 | Angiotensin-converting enzyme |
| DB00925 | Phenoxybenzamine | P35348 | Alpha-1A adrenergic receptor |
| DB00925 | Phenoxybenzamine | P08913 | Alpha-2A adrenergic receptor |
| DB00925 | Phenoxybenzamine | P18825 | Alpha-2C adrenergic receptor |
| DB00925 | Phenoxybenzamine | P18089 | Alpha-2B adrenergic receptor |
| DB00925 | Phenoxybenzamine | P0DP23 | Calmodulin |
| DB00925 | Phenoxybenzamine | P07550 | Beta-2 adrenergic receptor |
| DB00925 | Phenoxybenzamine | P35368 | Alpha-1B adrenergic receptor |
| DB00925 | Phenoxybenzamine | P25100 | Alpha-1D adrenergic receptor |
| DB00999 | Hydrochlorothiazide | P55017 | Solute carrier family 12 member 3 |
| DB00999 | Hydrochlorothiazide | P00915 | Carbonic anhydrase 1 |
| DB00999 | Hydrochlorothiazide | P00918 | Carbonic anhydrase 2 |
| DB00999 | Hydrochlorothiazide | Q12791 | Calcium-activated potassium channel subunit alpha-1 |
| DB01021 | Trichlormethiazide | P55017 | Solute carrier family 12 member 3 |
| DB01021 | Trichlormethiazide | P05023 | Sodium/potassium-transporting ATPase subunit alpha-1 |
| DB01021 | Trichlormethiazide | P00915 | Carbonic anhydrase 1 |
| DB01021 | Trichlormethiazide | P00918 | Carbonic anhydrase 2 |
| DB01021 | Trichlormethiazide | P22748 | Carbonic anhydrase 4 |
| DB01023 | Felodipine | Q13936 | Voltage-dependent L-type calcium channel subunit alpha-1C |
| DB01023 | Felodipine | P54289 | Voltage-dependent calcium channel subunit alpha-2/delta-1 |
| DB01023 | Felodipine | Q08289 | Voltage-dependent L-type calcium channel subunit beta-2 |
| DB01023 | Felodipine | Q01668 | Voltage-dependent L-type calcium channel subunit alpha-1D |
| DB01023 | Felodipine | Q13698 | Voltage-dependent L-type calcium channel subunit alpha-1S |
| DB01023 | Felodipine | O95180 | Voltage-dependent T-type calcium channel subunit alpha-1H |
| DB01023 | Felodipine | Q9NY47 | Voltage-dependent calcium channel subunit alpha-2/delta-2 |
| DB01023 | Felodipine | P0DP23 | Calmodulin |
| DB01023 | Felodipine | Q01064 | Calcium/calmodulin-dependent 3' |
| DB01023 | Felodipine | P54750 | Calcium/calmodulin-dependent 3' |
| DB01023 | Felodipine | P08235 | Mineralocorticoid receptor |
| DB01023 | Felodipine | P02585 | Troponin C |
| DB01023 | Felodipine | P63316 | Troponin C |
| DB01054 | Nitrendipine | Q13936 | Voltage-dependent L-type calcium channel subunit alpha-1C |
| DB01054 | Nitrendipine | P54289 | Voltage-dependent calcium channel subunit alpha-2/delta-1 |
| DB01054 | Nitrendipine | Q08289 | Voltage-dependent L-type calcium channel subunit beta-2 |
| DB01054 | Nitrendipine | Q06432 | Voltage-dependent calcium channel gamma-1 subunit |
| DB01054 | Nitrendipine | Q01668 | Voltage-dependent L-type calcium channel subunit alpha-1D |
| DB01054 | Nitrendipine | Q13698 | Voltage-dependent L-type calcium channel subunit alpha-1S |
| DB01054 | Nitrendipine | Q9NY47 | Voltage-dependent calcium channel subunit alpha-2/delta-2 |
| DB01054 | Nitrendipine | O95180 | Voltage-dependent T-type calcium channel subunit alpha-1H |
| DB01119 | Diazoxide | Q14654 | ATP-sensitive inward rectifier potassium channel 11 |
| DB01119 | Diazoxide | P00915 | Carbonic anhydrase 1 |
| DB01119 | Diazoxide | P00918 | Carbonic anhydrase 2 |
| DB01119 | Diazoxide | P05023 | Sodium/potassium-transporting ATPase subunit alpha-1 |
| DB01119 | Diazoxide | Q12791 | Calcium-activated potassium channel subunit alpha-1 |
| DB01119 | Diazoxide | P55017 | Solute carrier family 12 member 3 |
| DB01203 | Nadolol | P08588 | Beta-1 adrenergic receptor |
| DB01203 | Nadolol | P07550 | Beta-2 adrenergic receptor |
| DB01275 | Hydralazine | Q16853 | Membrane primary amine oxidase |
| DB01275 | Hydralazine | P13674 | Prolyl 4-hydroxylase subunit alpha-1 |
| DB01324 | Polythiazide | P55017 | Solute carrier family 12 member 3 |
| DB01340 | Cilazapril | P12821 | Angiotensin-converting enzyme |
| DB04840 | Debrisoquin | P23975 | Sodium-dependent noradrenaline transporter |
| DB06403 | Ambrisentan | P25101 | Endothelin-1 receptor |
| DB06403 | Ambrisentan | P24530 | Endothelin B receptor |
| DB08932 | Macitentan | P25101 | Endothelin-1 receptor |
| DB08932 | Macitentan | P24530 | Endothelin B receptor |
| DB09236 | Lacidipine | Q13936 | Voltage-dependent L-type calcium channel subunit alpha-1C |
| DB09236 | Lacidipine | Q01668 | Voltage-dependent L-type calcium channel subunit alpha-1D |
| DB09236 | Lacidipine | O60840 | Voltage-dependent L-type calcium channel subunit alpha-1F |
| DB09236 | Lacidipine | Q13698 | Voltage-dependent L-type calcium channel subunit alpha-1S |
| DB09236 | Lacidipine | Q02641 | Voltage-dependent L-type calcium channel subunit beta-1 |
| DB09236 | Lacidipine | Q08289 | Voltage-dependent L-type calcium channel subunit beta-2 |
| DB09236 | Lacidipine | P54284 | Voltage-dependent L-type calcium channel subunit beta-3 |
| DB09236 | Lacidipine | O00305 | Voltage-dependent L-type calcium channel subunit beta-4 |
| DB11362 | Selexipag | P43119 | Prostacyclin receptor |

| **g3** | | | |
| --- | --- | --- | --- |
| **DrugBank_ID** | **Name** | **UniProt_ID** | **Target Name** |
| DB00178 | Ramipril | P12821 | Angiotensin-converting enzyme |
| DB00178 | Ramipril | P46663 | B1 bradykinin receptor |
| DB00226 | Guanadrel | P23975 | Sodium-dependent noradrenaline transporter |
| DB00287 | Travoprost | P43088 | Prostaglandin F2-alpha receptor |
| DB00373 | Timolol | P08588 | Beta-1 adrenergic receptor |
| DB00373 | Timolol | P07550 | Beta-2 adrenergic receptor |
| DB00373 | Timolol | P00720 | Endolysin |
| DB00575 | Clonidine | P08913 | Alpha-2A adrenergic receptor |
| DB00575 | Clonidine | P18089 | Alpha-2B adrenergic receptor |
| DB00575 | Clonidine | P18825 | Alpha-2C adrenergic receptor |
| DB00575 | Clonidine | P35348 | Alpha-1A adrenergic receptor |
| DB00575 | Clonidine | P35368 | Alpha-1B adrenergic receptor |
| DB00575 | Clonidine | P25100 | Alpha-1D adrenergic receptor |
| DB00584 | Enalapril | P12821 | Angiotensin-converting enzyme |
| DB00654 | Latanoprost | P43088 | Prostaglandin F2-alpha receptor |
| DB00657 | Mecamylamine | Q15822 | Neuronal acetylcholine receptor subunit alpha-2 |
| DB00657 | Mecamylamine | P36544 | Neuronal acetylcholine receptor subunit alpha-7 |
| DB00657 | Mecamylamine | P43681 | Neuronal acetylcholine receptor subunit alpha-4 |
| DB00657 | Mecamylamine | P17787 | Neuronal acetylcholine receptor subunit beta-2 |
| DB00722 | Lisinopril | P12821 | Angiotensin-converting enzyme |
| DB00790 | Perindopril | P12821 | Angiotensin-converting enzyme |
| DB00790 | Perindopril | Q6FHJ7 | Secreted frizzled-related protein 4 |
| DB00797 | Tolazoline | P35348 | Alpha-1A adrenergic receptor |
| DB00797 | Tolazoline | P08913 | Alpha-2A adrenergic receptor |
| DB00797 | Tolazoline | P35367 | Histamine H1 receptor |
| DB00797 | Tolazoline | P25021 | Histamine H2 receptor |
| DB00797 | Tolazoline | P18825 | Alpha-2C adrenergic receptor |
| DB00797 | Tolazoline | P18089 | Alpha-2B adrenergic receptor |
| DB00869 | Dorzolamide | P00918 | Carbonic anhydrase 2 |
| DB00869 | Dorzolamide | P22748 | Carbonic anhydrase 4 |
| DB00869 | Dorzolamide | P00915 | Carbonic anhydrase 1 |
| DB00869 | Dorzolamide | P07451 | Carbonic anhydrase 3 |
| DB00876 | Eprosartan | P30556 | Type-1 angiotensin II receptor |
| DB00905 | Bimatoprost | P43088 | Prostaglandin F2-alpha receptor |
| DB00905 | Bimatoprost | P34995 | Prostaglandin E2 receptor EP1 subtype |
| DB00905 | Bimatoprost | P43115 | Prostaglandin E2 receptor EP3 subtype |
| DB00905 | Bimatoprost | P42330 | Aldo-keto reductase family 1 member C3 |
| DB00960 | Pindolol | P08588 | Beta-1 adrenergic receptor |
| DB00960 | Pindolol | P07550 | Beta-2 adrenergic receptor |
| DB00960 | Pindolol | P08908 | 5-hydroxytryptamine receptor 1A |
| DB00960 | Pindolol | P28222 | 5-hydroxytryptamine receptor 1B |
| DB00960 | Pindolol | P13945 | Beta-3 adrenergic receptor |
| DB01090 | Pentolinium | Q9GZZ6 | Neuronal acetylcholine receptor subunit alpha-10 |
| DB01090 | Pentolinium | P32297 | Neuronal acetylcholine receptor subunit alpha-3 |
| DB01090 | Pentolinium | P30926 | Neuronal acetylcholine receptor subunit beta-4 |
| DB01197 | Captopril | P12821 | Angiotensin-converting enzyme |
| DB01197 | Captopril | P08253 | 72 kDa type IV collagenase |
| DB01197 | Captopril | P14780 | Matrix metalloproteinase-9 |
| DB01197 | Captopril | P09960 | Leukotriene A-4 hydrolase |
| DB01197 | Captopril | P46663 | B1 bradykinin receptor |
| DB01240 | Epoprostenol | Q9H244 | P2Y purinoceptor 12 |
| DB01240 | Epoprostenol | P43119 | Prostacyclin receptor |
| DB01240 | Epoprostenol | Q16647 | Prostacyclin synthase |
| DB01348 | Spirapril | P12821 | Angiotensin-converting enzyme |
| DB01359 | Penbutolol | P08588 | Beta-1 adrenergic receptor |
| DB01359 | Penbutolol | P07550 | Beta-2 adrenergic receptor |
| DB01359 | Penbutolol | P08908 | 5-hydroxytryptamine receptor 1A |
| DB01359 | Penbutolol | P28222 | 5-hydroxytryptamine receptor 1B |
| DB04948 | Lofexidine | P08913 | Alpha-2A adrenergic receptor |
| DB04948 | Lofexidine | P35348 | Alpha-1A adrenergic receptor |
| DB04948 | Lofexidine | P08908 | 5-hydroxytryptamine receptor 1A |
| DB04948 | Lofexidine | P34969 | 5-hydroxytryptamine receptor 7 |
| DB04948 | Lofexidine | P28335 | 5-hydroxytryptamine receptor 2C |
| DB04948 | Lofexidine | P28221 | 5-hydroxytryptamine receptor 1D |
| DB09242 | Moxonidine | P08913 | Alpha-2A adrenergic receptor |
| DB09242 | Moxonidine | Q9Y2I1 | Nischarin |
| DB09477 | Enalaprilat | P12821 | Angiotensin-converting enzyme |
| DB09477 | Enalaprilat | P46663 | B1 bradykinin receptor |
| DB11738 | Rilmenidine | P08913 | Alpha-2A adrenergic receptor |

| **g4** | | | |
| --- | --- | --- | --- |
| **DrugBank_ID** | **Name** | **UniProt_ID** | **Target Name** |
| DB00206 | Reserpine | Q05940 | Synaptic vesicular amine transporter |
| DB00206 | Reserpine | P54219 | Chromaffin granule amine transporter |
| DB00206 | Reserpine | O15392 | Baculoviral IAP repeat-containing protein 5 |
| DB00457 | Prazosin | P35348 | Alpha-1A adrenergic receptor |
| DB00457 | Prazosin | P35368 | Alpha-1B adrenergic receptor |
| DB00457 | Prazosin | P25100 | Alpha-1D adrenergic receptor |
| DB00457 | Prazosin | Q12809 | Potassium voltage-gated channel subfamily H member 2 |
| DB00457 | Prazosin | Q9H252 | Potassium voltage-gated channel subfamily H member 6 |
| DB00457 | Prazosin | Q9NS40 | Potassium voltage-gated channel subfamily H member 7 |
| DB00457 | Prazosin | P08913 | Alpha-2A adrenergic receptor |
| DB00457 | Prazosin | P18089 | Alpha-2B adrenergic receptor |
| DB00528 | Lercanidipine | Q06432 | Voltage-dependent calcium channel gamma-1 subunit |
| DB00559 | Bosentan | P25101 | Endothelin-1 receptor |
| DB00559 | Bosentan | P24530 | Endothelin B receptor |
| DB00590 | Doxazosin | P35348 | Alpha-1A adrenergic receptor |
| DB00590 | Doxazosin | P35368 | Alpha-1B adrenergic receptor |
| DB00590 | Doxazosin | P25100 | Alpha-1D adrenergic receptor |
| DB00590 | Doxazosin | Q12809 | Potassium voltage-gated channel subfamily H member 2 |
| DB00590 | Doxazosin | Q9H252 | Potassium voltage-gated channel subfamily H member 6 |
| DB00590 | Doxazosin | Q9NS40 | Potassium voltage-gated channel subfamily H member 7 |
| DB00820 | Tadalafil | O76074 | cGMP-specific 3' |
| DB00820 | Tadalafil | Q9HCR9 | Dual 3' |
| DB01089 | Deserpidine | Q05940 | Synaptic vesicular amine transporter |
| DB01162 | Terazosin | P35348 | Alpha-1A adrenergic receptor |
| DB01162 | Terazosin | P35368 | Alpha-1B adrenergic receptor |
| DB01162 | Terazosin | P25100 | Alpha-1D adrenergic receptor |
| DB01162 | Terazosin | Q12809 | Potassium voltage-gated channel subfamily H member 2 |
| DB01162 | Terazosin | Q9H252 | Potassium voltage-gated channel subfamily H member 6 |
| DB01162 | Terazosin | Q9NS40 | Potassium voltage-gated channel subfamily H member 7 |
| DB01180 | Rescinnamine | P12821 | Angiotensin-converting enzyme |
| DB04861 | Nebivolol | P08588 | Beta-1 adrenergic receptor |
| DB04861 | Nebivolol | P07550 | Beta-2 adrenergic receptor |
| DB08931 | Riociguat | P33402 | Guanylate cyclase soluble subunit alpha-2 |
| DB09238 | Manidipine | Q13936 | Voltage-dependent L-type calcium channel subunit alpha-1C |
| DB09238 | Manidipine | Q01668 | Voltage-dependent L-type calcium channel subunit alpha-1D |
| DB09238 | Manidipine | O60840 | Voltage-dependent L-type calcium channel subunit alpha-1F |
| DB09238 | Manidipine | Q13698 | Voltage-dependent L-type calcium channel subunit alpha-1S |
| DB09238 | Manidipine | Q02641 | Voltage-dependent L-type calcium channel subunit beta-1 |
| DB09238 | Manidipine | Q08289 | Voltage-dependent L-type calcium channel subunit beta-2 |
| DB09238 | Manidipine | P54284 | Voltage-dependent L-type calcium channel subunit beta-3 |
| DB09238 | Manidipine | O00305 | Voltage-dependent L-type calcium channel subunit beta-4 |
| DB09238 | Manidipine | O43497 | Voltage-dependent T-type calcium channel subunit alpha-1G |
| DB09238 | Manidipine | O95180 | Voltage-dependent T-type calcium channel subunit alpha-1H |
| DB09238 | Manidipine | Q9P0X4 | Voltage-dependent T-type calcium channel subunit alpha-1I |

| **g5** | | | |
| --- | --- | --- | --- |
| **DrugBank_ID** | **Name** | **UniProt_ID** | **Target Name** |
| DB00177 | Valsartan | P30556 | Type-1 angiotensin II receptor |
| DB00270 | Isradipine | Q13936 | Voltage-dependent L-type calcium channel subunit alpha-1C |
| DB00270 | Isradipine | P54289 | Voltage-dependent calcium channel subunit alpha-2/delta-1 |
| DB00270 | Isradipine | Q08289 | Voltage-dependent L-type calcium channel subunit beta-2 |
| DB00270 | Isradipine | O95180 | Voltage-dependent T-type calcium channel subunit alpha-1H |
| DB00270 | Isradipine | Q9NY47 | Voltage-dependent calcium channel subunit alpha-2/delta-2 |
| DB00270 | Isradipine | Q01668 | Voltage-dependent L-type calcium channel subunit alpha-1D |
| DB00270 | Isradipine | Q13698 | Voltage-dependent L-type calcium channel subunit alpha-1S |
| DB00310 | Chlorthalidone | Q13621 | Solute carrier family 12 member 1 |
| DB00343 | Diltiazem | Q06432 | Voltage-dependent calcium channel gamma-1 subunit |
| DB00374 | Treprostinil | P43119 | Prostacyclin receptor |
| DB00374 | Treprostinil | Q03181 | Peroxisome proliferator-activated receptor delta |
| DB00374 | Treprostinil | Q9H244 | P2Y purinoceptor 12 |
| DB00492 | Fosinopril | P12821 | Angiotensin-converting enzyme |
| DB00519 | Trandolapril | P12821 | Angiotensin-converting enzyme |
| DB00542 | Benazepril | P12821 | Angiotensin-converting enzyme |
| DB00606 | Cyclothiazide | P54710 | Sodium/potassium-transporting ATPase subunit gamma |
| DB00606 | Cyclothiazide | P00915 | Carbonic anhydrase 1 |
| DB00606 | Cyclothiazide | P00918 | Carbonic anhydrase 2 |
| DB00606 | Cyclothiazide | P22748 | Carbonic anhydrase 4 |
| DB00606 | Cyclothiazide | Q6FHJ7 | Secreted frizzled-related protein 4 |
| DB00692 | Phentolamine | P08913 | Alpha-2A adrenergic receptor |
| DB00692 | Phentolamine | P35348 | Alpha-1A adrenergic receptor |
| DB00692 | Phentolamine | P35368 | Alpha-1B adrenergic receptor |
| DB00692 | Phentolamine | P25100 | Alpha-1D adrenergic receptor |
| DB00800 | Fenoldopam | P21918 | D(1B) dopamine receptor |
| DB00800 | Fenoldopam | P21728 | D(1A) dopamine receptor |
| DB00800 | Fenoldopam | P18089 | Alpha-2B adrenergic receptor |
| DB00800 | Fenoldopam | P18825 | Alpha-2C adrenergic receptor |
| DB00800 | Fenoldopam | P08913 | Alpha-2A adrenergic receptor |
| DB00800 | Fenoldopam | P35368 | Alpha-1B adrenergic receptor |
| DB00800 | Fenoldopam | P25100 | Alpha-1D adrenergic receptor |
| DB00800 | Fenoldopam | P35348 | Alpha-1A adrenergic receptor |
| DB00808 | Indapamide | P55017 | Solute carrier family 12 member 3 |
| DB01136 | Carvedilol | P08588 | Beta-1 adrenergic receptor |
| DB01136 | Carvedilol | P35348 | Alpha-1A adrenergic receptor |
| DB01136 | Carvedilol | O95298 | NADH dehydrogenase [ubiquinone] 1 subunit C2 |
| DB01136 | Carvedilol | P07550 | Beta-2 adrenergic receptor |
| DB01136 | Carvedilol | P15692 | Vascular endothelial growth factor A |
| DB01136 | Carvedilol | P16860 | Natriuretic peptides B |
| DB01136 | Carvedilol | P17302 | Gap junction alpha-1 protein |
| DB01136 | Carvedilol | Q12809 | Potassium voltage-gated channel subfamily H member 2 |
| DB01136 | Carvedilol | P19320 | Vascular cell adhesion protein 1 |
| DB01136 | Carvedilol | P25100 | Alpha-1D adrenergic receptor |
| DB01136 | Carvedilol | P35368 | Alpha-1B adrenergic receptor |
| DB01136 | Carvedilol | P18825 | Alpha-2C adrenergic receptor |
| DB01136 | Carvedilol | P18089 | Alpha-2B adrenergic receptor |
| DB01136 | Carvedilol | P08913 | Alpha-2A adrenergic receptor |
| DB01136 | Carvedilol | P16581 | E-selectin |
| DB01136 | Carvedilol | Q16665 | Hypoxia-inducible factor 1-alpha |
| DB01136 | Carvedilol | P48050 | Inward rectifier potassium channel 4 |
| DB01244 | Bepridil | O00555 | Voltage-dependent P/Q-type calcium channel subunit alpha-1A |
| DB01244 | Bepridil | O95180 | Voltage-dependent T-type calcium channel subunit alpha-1H |
| DB01244 | Bepridil | Q9NY47 | Voltage-dependent calcium channel subunit alpha-2/delta-2 |
| DB01244 | Bepridil | P05023 | Sodium/potassium-transporting ATPase subunit alpha-1 |
| DB01244 | Bepridil | P51787 | Potassium voltage-gated channel subfamily KQT member 1 |
| DB01244 | Bepridil | P63316 | Troponin C |
| DB01244 | Bepridil | P0DP23 | Calmodulin |
| DB01244 | Bepridil | Q01064 | Calcium/calmodulin-dependent 3' |
| DB01244 | Bepridil | P54750 | Calcium/calmodulin-dependent 3' |
| DB01244 | Bepridil | Q12809 | Potassium voltage-gated channel subfamily H member 2 |

| **g6** | | | |
| --- | --- | --- | --- |
| **DrugBank_ID** | **Name** | **UniProt_ID** | **Target Name** |
| DB00187 | Esmolol | P08588 | Beta-1 adrenergic receptor |
| DB00195 | Betaxolol | P08588 | Beta-1 adrenergic receptor |
| DB00195 | Betaxolol | P07550 | Beta-2 adrenergic receptor |
| DB00217 | Bethanidine | P08913 | Alpha-2A adrenergic receptor |
| DB00217 | Bethanidine | P18089 | Alpha-2B adrenergic receptor |
| DB00217 | Bethanidine | P18825 | Alpha-2C adrenergic receptor |
| DB00217 | Bethanidine | P48048 | ATP-sensitive inward rectifier potassium channel 1 |
| DB00217 | Bethanidine | P08588 | Beta-1 adrenergic receptor |
| DB00217 | Bethanidine | P07550 | Beta-2 adrenergic receptor |
| DB00217 | Bethanidine | P13945 | Beta-3 adrenergic receptor |
| DB00264 | Metoprolol | P08588 | Beta-1 adrenergic receptor |
| DB00264 | Metoprolol | P07550 | Beta-2 adrenergic receptor |
| DB00325 | Nitroprusside | P16066 | Atrial natriuretic peptide receptor 1 |
| DB00335 | Atenolol | P08588 | Beta-1 adrenergic receptor |
| DB00335 | Atenolol | P07550 | Beta-2 adrenergic receptor |
| DB00612 | Bisoprolol | P08588 | Beta-1 adrenergic receptor |
| DB00612 | Bisoprolol | P07550 | Beta-2 adrenergic receptor |
| DB00629 | Guanabenz | P08913 | Alpha-2A adrenergic receptor |
| DB00629 | Guanabenz | P18089 | Alpha-2B adrenergic receptor |
| DB00727 | Nitroglycerin | P16066 | Atrial natriuretic peptide receptor 1 |
| DB00765 | Metyrosine | P07101 | Tyrosine 3-monooxygenase |
| DB00968 | Methyldopa | P08913 | Alpha-2A adrenergic receptor |
| DB00968 | Methyldopa | P20711 | Aromatic-L-amino-acid decarboxylase |
| DB01018 | Guanfacine | P08913 | Alpha-2A adrenergic receptor |
| DB01018 | Guanfacine | P18089 | Alpha-2B adrenergic receptor |
| DB01158 | Bretylium | P05023 | Sodium/potassium-transporting ATPase subunit alpha-1 |
| DB01170 | Guanethidine | P23975 | Sodium-dependent noradrenaline transporter |
| DB01193 | Acebutolol | P08588 | Beta-1 adrenergic receptor |
| DB01193 | Acebutolol | P07550 | Beta-2 adrenergic receptor |
| DB01214 | Metipranolol | P07550 | Beta-2 adrenergic receptor |
| DB01214 | Metipranolol | P08588 | Beta-1 adrenergic receptor |
| DB01297 | Practolol | P08588 | Beta-1 adrenergic receptor |
| DB01580 | Oxprenolol | P08588 | Beta-1 adrenergic receptor |
| DB01580 | Oxprenolol | P07550 | Beta-2 adrenergic receptor |
| DB01580 | Oxprenolol | P13945 | Beta-3 adrenergic receptor |
| DB01626 | Pargyline | P27338 | Amine oxidase [flavin-containing] B |
| DB01626 | Pargyline | P21397 | Amine oxidase [flavin-containing] A |
| DB04846 | Celiprolol | P08588 | Beta-1 adrenergic receptor |
| DB04846 | Celiprolol | P07550 | Beta-2 adrenergic receptor |
| DB04846 | Celiprolol | P13945 | Beta-3 adrenergic receptor |
| DB04846 | Celiprolol | P08913 | Alpha-2A adrenergic receptor |
| DB04846 | Celiprolol | P18089 | Alpha-2B adrenergic receptor |
| DB04846 | Celiprolol | P18825 | Alpha-2C adrenergic receptor |
| DB09026 | Aliskiren | P00797 | Renin |
| DB09220 | Nicorandil | O60706 | ATP-binding cassette sub-family C member 9 |

**Table S3.** List of the unique targets and tc-genes IDs associated with groups.

| **g1** | |  | **g2** | |  | **g3** | |  | **g4** | |  | **g5** | |  | **g6** | |
| --- | --- | --- | --- | --- | --- | --- | --- | --- | --- | --- | --- | --- | --- | --- | --- | --- |
| **UniProt_ID** | **Gene_ID** |  | **UniProt_ID** | **Gene_ID** |  | **UniProt_ID** | **Gene_ID** |  | **UniProt_ID** | **Gene_ID** |  | **UniProt_ID** | **Gene_ID** |  | **UniProt_ID** | **Gene_ID** |
| P05412 | JUN |  | O00305 | CACNB4 |  | P00720 | E |  | O00305 | CACNB4 |  | O00555 | CACNA1A |  | O60706 | ABCC9 |
| P08235 | NR3C2 |  | O43570 | CA12 |  | P00915 | CA1 |  | O15392 | BIRC5 |  | O95180 | CACNA1H |  | P00797 | REN |
| P24530 | EDNRB |  | O60840 | CACNA1F |  | P00918 | CA2 |  | O43497 | CACNA1G |  | O95298 | NDUFC2 |  | P05023 | ATP1A1 |
| P25101 | EDNRA |  | O95180 | CACNA1H |  | P07451 | CA3 |  | O60840 | CACNA1F |  | P00915 | CA1 |  | P07101 | TH |
| P30556 | AGTR1 |  | P00797 | REN |  | P07550 | ADRB2 |  | O76074 | PDE5A |  | P00918 | CA2 |  | P07550 | ADRB2 |
| P37231 | PPARG |  | P00915 | CA1 |  | P08253 | MMP2 |  | O95180 | CACNA1H |  | P05023 | ATP1A1 |  | P08588 | ADRB1 |
| Q9GZZ6 | CHRNA10 |  | P00918 | CA2 |  | P08588 | ADRB1 |  | P07550 | ADRB2 |  | P07550 | ADRB2 |  | P08913 | ADRA2A |
|  |  |  | P02585 | TNNC2 |  | P08908 | HTR1A |  | P08588 | ADRB1 |  | P08588 | ADRB1 |  | P13945 | ADRB3 |
|  |  |  | P05023 | ATP1A1 |  | P08913 | ADRA2A |  | P08913 | ADRA2A |  | P08913 | ADRA2A |  | P16066 | NPR1 |
|  |  |  | P07550 | ADRB2 |  | P09960 | LTA4H |  | P12821 | ACE |  | P0DP23 | CALM1 |  | P18089 | ADRA2B |
|  |  |  | P08172 | CHRM2 |  | P12821 | ACE |  | P18089 | ADRA2B |  | P12821 | ACE |  | P18825 | ADRA2C |
|  |  |  | P08173 | CHRM4 |  | P13945 | ADRB3 |  | P24530 | EDNRB |  | P15692 | VEGFA |  | P20711 | DDC |
|  |  |  | P08235 | NR3C2 |  | P14780 | MMP9 |  | P25100 | ADRA1D |  | P16581 | SELE |  | P21397 | MAOA |
|  |  |  | P08588 | ADRB1 |  | P17787 | CHRNB2 |  | P25101 | EDNRA |  | P16860 | NPPB |  | P23975 | SLC6A2 |
|  |  |  | P08908 | HTR1A |  | P18089 | ADRA2B |  | P33402 | GUCY1A2 |  | P17302 | GJA1 |  | P27338 | MAOB |
|  |  |  | P08912 | CHRM5 |  | P18825 | ADRA2C |  | P35348 | ADRA1A |  | P18089 | ADRA2B |  | P48048 | KCNJ1 |
|  |  |  | P08913 | ADRA2A |  | P22748 | CA4 |  | P35368 | ADRA1B |  | P18825 | ADRA2C |  |  |  |
|  |  |  | P0DP23 | CALM1 |  | P23975 | SLC6A2 |  | P54219 | SLC18A1 |  | P19320 | VCAM1 |  |  |  |
|  |  |  | P11229 | CHRM1 |  | P25021 | HRH2 |  | P54284 | CACNB3 |  | P21728 | DRD1 |  |  |  |
|  |  |  | P12821 | ACE |  | P25100 | ADRA1D |  | Q01668 | CACNA1D |  | P21918 | DRD5 |  |  |  |
|  |  |  | P13674 | P4HA1 |  | P28221 | HTR1D |  | Q02641 | CACNB1 |  | P22748 | CA4 |  |  |  |
|  |  |  | P13945 | ADRB3 |  | P28222 | HTR1B |  | Q05940 | SLC18A2 |  | P25100 | ADRA1D |  |  |  |
|  |  |  | P17405 | SMPD1 |  | P28335 | HTR2C |  | Q06432 | CACNG1 |  | P30556 | AGTR1 |  |  |  |
|  |  |  | P18089 | ADRA2B |  | P30556 | AGTR1 |  | Q08289 | CACNB2 |  | P35348 | ADRA1A |  |  |  |
|  |  |  | P18825 | ADRA2C |  | P30926 | CHRNB4 |  | Q12809 | KCNH2 |  | P35368 | ADRA1B |  |  |  |
|  |  |  | P20309 | CHRM3 |  | P32297 | CHRNA3 |  | Q13698 | CACNA1S |  | P43119 | PTGIR |  |  |  |
|  |  |  | P22748 | CA4 |  | P34969 | HTR7 |  | Q13936 | CACNA1C |  | P48050 | KCNJ4 |  |  |  |
|  |  |  | P23219 | PTGS1 |  | P34995 | PTGER1 |  | Q9H252 | KCNH6 |  | P51787 | KCNQ1 |  |  |  |
|  |  |  | P23975 | SLC6A2 |  | P35348 | ADRA1A |  | Q9HCR9 | PDE11A |  | P54289 | CACNA2D1 |  |  |  |
|  |  |  | P24530 | EDNRB |  | P35367 | HRH1 |  | Q9NS40 | KCNH7 |  | P54710 | FXYD2 |  |  |  |
|  |  |  | P25100 | ADRA1D |  | P35368 | ADRA1B |  | Q9P0X4 | CACNA1I |  | P54750 | PDE1A |  |  |  |
|  |  |  | P25101 | EDNRA |  | P36544 | CHRNA7 |  |  |  |  | P55017 | SLC12A3 |  |  |  |
|  |  |  | P28222 | HTR1B |  | P42330 | AKR1C3 |  |  |  |  | P63316 | TNNC1 |  |  |  |
|  |  |  | P35348 | ADRA1A |  | P43088 | PTGFR |  |  |  |  | Q01064 | PDE1B |  |  |  |
|  |  |  | P35368 | ADRA1B |  | P43115 | PTGER3 |  |  |  |  | Q01668 | CACNA1D |  |  |  |
|  |  |  | P35869 | AHR |  | P43119 | PTGIR |  |  |  |  | Q03181 | PPARD |  |  |  |
|  |  |  | P43119 | PTGIR |  | P43681 | CHRNA4 |  |  |  |  | Q06432 | CACNG1 |  |  |  |
|  |  |  | P48048 | KCNJ1 |  | P46663 | BDKRB1 |  |  |  |  | Q08289 | CACNB2 |  |  |  |
|  |  |  | P54284 | CACNB3 |  | Q15822 | CHRNA2 |  |  |  |  | Q12809 | KCNH2 |  |  |  |
|  |  |  | P54289 | CACNA2D1 |  | Q16647 | PTGIS |  |  |  |  | Q13621 | SLC12A1 |  |  |  |
|  |  |  | P54750 | PDE1A |  | Q6FHJ7 | SFRP4 |  |  |  |  | Q13698 | CACNA1S |  |  |  |
|  |  |  | P55017 | SLC12A3 |  | Q9GZZ6 | CHRNA10 |  |  |  |  | Q13936 | CACNA1C |  |  |  |
|  |  |  | P63316 | TNNC1 |  | Q9H244 | P2RY12 |  |  |  |  | Q16665 | HIF1A |  |  |  |
|  |  |  | Q00975 | CACNA1B |  | Q9Y2I1 | NISCH |  |  |  |  | Q6FHJ7 | SFRP4 |  |  |  |
|  |  |  | Q01064 | PDE1B |  |  |  |  |  |  |  | Q9H244 | P2RY12 |  |  |  |
|  |  |  | Q01668 | CACNA1D |  |  |  |  |  |  |  | Q9NY47 | CACNA2D2 |  |  |  |
|  |  |  | Q02641 | CACNB1 |  |  |  |  |  |  |  |  |  |  |  |  |
|  |  |  | Q06432 | CACNG1 |  |  |  |  |  |  |  |  |  |  |  |  |
|  |  |  | Q08289 | CACNB2 |  |  |  |  |  |  |  |  |  |  |  |  |
|  |  |  | Q12791 | KCNMA1 |  |  |  |  |  |  |  |  |  |  |  |  |
|  |  |  | Q13621 | SLC12A1 |  |  |  |  |  |  |  |  |  |  |  |  |
|  |  |  | Q13698 | CACNA1S |  |  |  |  |  |  |  |  |  |  |  |  |
|  |  |  | Q13936 | CACNA1C |  |  |  |  |  |  |  |  |  |  |  |  |
|  |  |  | Q14654 | KCNJ11 |  |  |  |  |  |  |  |  |  |  |  |  |
|  |  |  | Q16790 | CA9 |  |  |  |  |  |  |  |  |  |  |  |  |
|  |  |  | Q16853 | AOC3 |  |  |  |  |  |  |  |  |  |  |  |  |
|  |  |  | Q6TME4 | CACNB1 |  |  |  |  |  |  |  |  |  |  |  |  |
|  |  |  | Q8IZS8 | CACNA2D3 |  |  |  |  |  |  |  |  |  |  |  |  |
|  |  |  | Q9BYF1 | ACE2 |  |  |  |  |  |  |  |  |  |  |  |  |
|  |  |  | Q9NY47 | CACNA2D2 |  |  |  |  |  |  |  |  |  |  |  |  |
|  |  |  |  |  |  |  |  |  |  |  |  |  |  |  |  |  |

**Table S4.** List of the common tc-genes among tc-genes2, tc-genes4 and tc-genes5.

| **Interacting Groups** | **No. of Common tc-genes** | **UniProt_ID tc-gene_ID** |
| --- | --- | --- |
| tc-genes2  tc-genes4  tc-genes5 | 14 | | O95180 CACNA1H | | --- | | P07550 ADRB2 | | P08588 ADRB1 | | P08913 ADRA2A | | P12821 ACE | | P18089 ADRA2B | | P25100 ADRA1D | | P35348 ADRA1A | | P35368 ADRA1B | | Q01668 CACNA1D | | Q06432 CACNG1 | | Q08289 CACNB2 | | Q13698 CACNA1S | | Q13936 CACNA1C | |

**Table S5.** Top 20 health conditions associated with tc-genesx (p < 2.0E-9).

g1

| Disease | Disease full name | Relevance (p-value) | Number of associated tc-genes1 | All tc-genes1 |
| --- | --- | --- | --- | --- |
| renal hypertension | renal hypertension | 2.00E-11 | 5 | NR3C2 AGTR1 JUN EDNRB PPARG |
| Autosomal dominant polycystic ... | Autosomal dominant polycystic kidney disease | 2.00E-11 | 5 | AGTR1 PPARG JUN EDNRB EDNRA |
| secondary hypertension | secondary hypertension | 3.00E-11 | 5 | NR3C2 AGTR1 JUN EDNRB PPARG |
| primary hypertension | primary hypertension | 6.00E-11 | 6 | AGTR1 NR3C2 PPARG EDNRA JUN EDNRB |
| polycystic kidney disease | polycystic kidney disease | 9.00E-11 | 5 | AGTR1 EDNRA PPARG JUN EDNRB |
| aneurysm | aneurysm | 1.00E-10 | 6 | PPARG AGTR1 EDNRA JUN NR3C2 EDNRB |
| neointimal hyperplasia | neointimal hyperplasia | 1.00E-10 | 6 | PPARG AGTR1 EDNRB JUN NR3C2 EDNRA |
| Polycystic Kidney Disease | Polycystic Kidney Disease | 2.00E-10 | 5 | AGTR1 PPARG JUN EDNRA EDNRB |
| diastolic heart failure | diastolic heart failure | 2.00E-10 | 2 | NR3C2 AGTR1 |
| Paramyxoviridae infectious dis... | Paramyxoviridae infectious disease | 3.00E-10 | 6 | NR3C2 JUN EDNRB AGTR1 PPARG EDNRA |
| malignant hypertension | malignant hypertension | 3.00E-10 | 4 | AGTR1 PPARG EDNRB NR3C2 |
| brain ischemia | brain ischemia | 5.00E-10 | 6 | PPARG AGTR1 NR3C2 EDNRB EDNRA JUN |
| abortion | abortion | 9.00E-10 | 6 | PPARG NR3C2 EDNRB EDNRA JUN AGTR1 |
| endothelial dysfunction | endothelial dysfunction | 1E-09 | 6 | AGTR1 EDNRA PPARG NR3C2 EDNRB JUN |
| Abnormality of cardiovascular ... | Abnormality of cardiovascular system morphology | 1E-09 | 6 | NR3C2 PPARG AGTR1 EDNRA EDNRB JUN |
| acute lung injury | acute lung injury | 1E-09 | 6 | PPARG JUN AGTR1 EDNRA NR3C2 EDNRB |
| ischemia reperfusion injury | ischemia reperfusion injury | 2E-09 | 6 | PPARG NR3C2 AGTR1 JUN EDNRB EDNRA |
| nephrotic syndrome | nephrotic syndrome | 2E-09 | 6 | NR3C2 PPARG EDNRA EDNRB JUN AGTR1 |
| Mononegavirales infectious dis... | Mononegavirales infectious disease | 2E-09 | 6 | AGTR1 NR3C2 PPARG JUN EDNRB EDNRA |
| leiomyoma | leiomyoma | 2E-09 | 6 | PPARG EDNRB AGTR1 EDNRA NR3C2 JUN |

| g2 | | | | |
| --- | --- | --- | --- | --- |
| Disease | Disease full name | Relevance (p-value) | Number of associated tc-genes2 | All tc-genes2 |
| abnormality of the urinary sys... | abnormality of the urinary system physiology | 3.00E-61 | 41 | SLC12A1 CHRM3 ADRA2A ADRA1D CHRM2 ADRA1B ADRA1A ADRA2C ADRA2B NR3C2 PDE1A PDE1B TNNC1 CACNB1 CACNA1D CACNB3 CACNA1B CACNA1H CACNA1F CACNA2D2 CACNA1C CACNA2D1 CACNB4 CACNG1 CACNB2 CACNA1S CACNA2D3 ADRB1 ADRB3 ADRB2 CA2 REN ACE ACE2 PTGS1 CA9 SLC12A3 EDNRB KCNJ1 HTR1A KCNJ11 |
| abnormal renal physiology | abnormal renal physiology | 4.00E-59 | 40 | SLC12A1 ADRA2B ADRA2A ADRA2C NR3C2 PDE1A TNNC1 PDE1B CACNA1D CACNB4 CACNA2D2 CACNA2D3 CACNA2D1 CACNA1F CACNB2 CACNG1 CACNB3 CACNA1C CACNB1 CACNA1H CACNA1B CACNA1S CHRM3 ADRB1 ADRB2 ADRA1D ADRB3 ADRA1A ADRA1B CA2 REN ACE ACE2 PTGS1 CA9 SLC12A3 EDNRB KCNJ1 HTR1A KCNJ11 |
| anxiety | anxiety | 5.00E-50 | 33 | HTR1A ADRA2A CACNA2D1 SLC6A2 ADRA2B ADRA2C ADRB3 CACNA1B ADRA1B ADRA1A ADRA1D ADRB1 ADRB2 CACNG1 CACNB4 CACNB1 CACNA1C CACNA1F CACNB3 CACNB2 CACNA2D2 CACNA1H CACNA2D3 CACNA1S CACNA1D HTR1B CA2 CA4 CHRM1 CA1 ACE2 SMPD1 REN |
| behavioral abnormality | behavioral abnormality | 4.00E-42 | 44 | HTR1A ADRB3 ADRA2A ADRA2C SLC6A2 ADRA2B CHRM1 CACNA2D1 CACNA1B ADRA1B ADRA1A ADRA1D ADRB2 ADRB1 CACNA1C CACNB2 CACNA2D2 CACNB1 CACNA1S CACNA2D3 CACNB3 CACNA1F CACNB4 CACNA1D CACNA1H CACNG1 HTR1B CA2 CA4 AAAS CHRM3 PTGS1 REN ACE CA1 NR3C2 SMPD1 EDNRA ACE2 KCNMA1 EDNRB CA9 KCNJ11 CHRM4 |
| Renal insufficiency | Renal insufficiency | 2.00E-41 | 25 | CACNA1B CACNA2D3 CACNB4 CACNG1 CACNA1H CHRM3 CACNB2 CACNA2D1 CACNA2D2 CACNA1D CACNA1F CACNA1C CACNB3 CACNA1S CACNB1 ADRA2B ADRA2A ADRA1B ADRA2C ADRB1 ADRA1D ADRB3 ADRB2 ADRA1A KCNJ11 |
| Sleep Disorder | Sleep Disorder | 2.00E-40 | 39 | SLC12A3 CA1 PTGS1 SLC6A2 CA12 ADRA2A CACNA2D1 ADRA2C CA2 ADRA2B CA4 HTR1A CACNA1D CACNA1C CACNA2D3 CACNA2D2 CACNA1F CACNB4 CACNB2 CACNB1 CACNA1B CACNA1S CACNA1H CACNG1 CACNB3 ADRB2 ADRB1 CHRM3 CHRM2 REN ACE NR3C2 HTR1B KCNJ11 AHR ADRB3 EDNRA KCNMA1 CA9 |
| cardioectodermal syndrome | cardioectodermal syndrome | 3.00E-40 | 39 | SLC12A3 CA1 PTGS1 SLC6A2 ADRA2A CA12 CA2 CA4 CACNA2D1 ADRA2C ADRA2B HTR1A CACNA1D CACNA1C CACNB2 CACNA1S CACNA1F CACNA2D2 CACNB4 CACNB1 CACNG1 CACNA1H CACNA1B CACNB3 CACNA2D3 ADRB2 ADRB1 CHRM2 CHRM3 REN ACE NR3C2 HTR1B KCNJ11 AHR ADRB3 EDNRA KCNMA1 CA9 |
| abnormality of the nervous sys... | abnormality of the nervous system | 7.00E-39 | 55 | CA2 CA1 CHRM1 CACNA1C PTGS1 CA4 HTR1A NR3C2 ADRB3 ADRB2 SLC6A2 ADRA2A CACNA1D ADRA2B CACNA2D1 ADRB1 CACNA1S CACNA1B ADRA2C CHRM2 CHRM3 ADRA1B ADRA1D CACNA1F CA12 ADRA1A HTR1B CACNA1H CACNA2D2 CACNA2D3 CACNB4 CACNB2 CACNG1 CACNB1 CACNB3 AAAS EDNRA KCNMA1 SLC12A1 SLC12A3 ACE2 REN ACE EDNRB SMPD1 AHR KCNJ11 ATP1A1 PDE1A KCNJ1 CA9 CALM1 CHRM5 PDE1B CHRM4 |
| anxiety disorder | anxiety disorder | 1.00E-38 | 45 | ADRB1 HTR1A CACNA1C SLC6A2 ADRB2 CA4 ADRA2A ADRA2C ADRA2B CACNB2 CACNA1B ADRA1B ADRA1D CACNA1F CACNB3 CACNG1 CA2 CACNA1H CACNA2D2 CACNB4 ADRA1A CACNA1D CACNA1S CACNA2D3 CACNA2D1 CACNB1 ADRB3 EDNRB CA1 SMPD1 HTR1B ACE CHRM1 REN ACE2 NR3C2 KCNMA1 ATP1A1 PDE1B EDNRA PDE1A PTGS1 CHRM3 CALM1 KCNJ11 |
| hypotension | hypotension | 1.00E-38 | 34 | ADRB2 ADRB1 SLC6A2 ADRA1B ADRA2A ADRA2C ADRA2B ADRA1A ADRB3 ADRA1D PTGS1 CA1 CA2 CA4 CA12 CACNA1C CACNA1D CACNA1F CACNA1S REN ACE HTR1A ACE2 SLC12A3 AHR HTR1B EDNRB SLC12A1 KCNMA1 EDNRA NR3C2 PTGIR KCNJ1 CACNB2 |
| drug-induced mental disorder | drug-induced mental disorder | 5.00E-37 | 47 | HTR1A PTGS1 ADRB2 SLC6A2 ADRA2A CACNA1B CHRM1 ADRA2C ADRB1 CA4 ADRA2B ADRA1B ADRA1A ADRA1D CA2 CACNA2D1 CHRM3 CACNA2D3 ADRB3 CACNA1D CACNA1C CACNA1F CACNA1S NR3C2 HTR1B ACE ATP1A1 REN CHRM2 SMPD1 AHR KCNMA1 CA9 AOC3 CA1 ACE2 KCNJ11 SLC12A3 EDNRA EDNRB CACNB4 CHRM5 CHRM4 CA12 TNNC2 CALM1 CACNB2 |
| drug dependence | drug dependence | 5.00E-37 | 47 | HTR1A PTGS1 ADRB2 SLC6A2 ADRA2A CACNA1B CHRM1 ADRA2C ADRB1 CA4 ADRA2B CA2 ADRA1A ADRA1D CACNA2D1 ADRA1B CHRM3 CACNA2D3 ADRB3 CACNA1D CACNA1C CACNA1F CACNA1S NR3C2 HTR1B ACE ATP1A1 REN CHRM2 SMPD1 AHR KCNMA1 CA9 AOC3 CA1 ACE2 KCNJ11 SLC12A3 EDNRA EDNRB CHRM5 CACNB4 CHRM4 CA12 TNNC2 CALM1 CACNB2 |
| Genetic neuromuscular disease | Genetic neuromuscular disease | 3.00E-36 | 54 | CACNA1S ATP1A1 ACE ADRA1B ADRB3 ADRB2 ADRB1 ADRA1D ADRA1A TNNC1 CA2 NR3C2 CA1 CA4 CA12 KCNMA1 SLC12A1 REN SMPD1 ACE2 EDNRA EDNRB CACNA2D2 CACNB4 CACNA1D CACNA1C KCNJ11 CACNB1 ADRA2B CHRM3 KCNJ1 CACNA1F PTGS1 AHR CACNA1B ADRA2A TNNC2 SLC6A2 P4HA1 CACNA2D3 CACNA1H PDE1B PDE1A CACNA2D1 HTR1A HTR1B PTGIR SLC12A3 CA9 AOC3 AAAS CHRM2 CACNG1 CACNB2 |
| neuromuscular disease | neuromuscular disease | 4.00E-36 | 55 | CACNA1S ATP1A1 ACE ADRA1B ADRB3 ADRB2 ADRB1 ADRA1A ADRA1D CA2 NR3C2 CA1 TNNC2 CA4 CA12 TNNC1 KCNMA1 SLC12A1 REN SMPD1 ACE2 EDNRA EDNRB CACNA2D2 CACNB4 CACNA1D KCNJ11 CACNB1 ADRA2B CHRM3 CACNA1C KCNJ1 CACNA1F PTGS1 AHR CACNA1B ADRA2A SLC6A2 P4HA1 CACNA2D3 PDE1B PDE1A HTR1A CACNA1H CACNA2D1 HTR1B SLC12A3 PTGIR CA9 AAAS CHRM1 AOC3 CHRM2 CACNG1 CACNB2 |
| primary hypertension | primary hypertension | 7.00E-36 | 28 | REN ACE ADRB1 SLC12A3 NR3C2 CACNA1C CACNA1D CACNA1F CACNA1S ADRB2 ADRB3 ADRA1B ADRA1D ADRA1A ATP1A1 KCNJ11 ACE2 SLC6A2 CACNB2 EDNRA ADRA2B KCNMA1 SLC12A1 EDNRB CA1 AOC3 AHR HTR1B |
| melanocytic skin neoplasm | melanocytic skin neoplasm | 2.00E-35 | 45 | CALM1 ADRA2C ADRA2A HTR1B HTR1A CACNA1D CHRM3 ADRB2 ADRB1 ATP1A1 KCNJ11 CACNB2 CACNA2D2 CACNB4 CACNB1 CACNA1B CACNA2D1 CACNB3 CACNA2D3 SLC6A2 ADRA2B ADRA1B ADRA1D ADRA1A CHRM5 CHRM2 CHRM4 CHRM1 CA1 AHR CA2 CA4 ACE REN ACE2 PDE1A PDE1B PTGS1 EDNRA CACNA1S AOC3 EDNRB KCNMA1 SLC12A3 CA9 |
| substance-related disorder | substance-related disorder | 6.00E-35 | 51 | HTR1A PTGS1 ADRB2 CA2 SLC6A2 ADRA2A CACNA1B NR3C2 ADRB1 CA4 ADRA2C CHRM1 ADRA1B ADRA1A ADRA2B PDE1A CACNA2D1 PDE1B ADRA1D CHRM3 CACNA2D3 ADRB3 CACNA1F CACNA1D CACNA1S CACNA1C HTR1B KCNMA1 ACE ATP1A1 ACE2 REN SMPD1 CHRM2 AHR CA1 CA9 AOC3 KCNJ11 SLC12A3 EDNRA EDNRB KCNJ1 CHRM5 CACNB4 SLC12A1 CHRM4 CA12 TNNC2 CALM1 CACNB2 |
| nicotine dependence | nicotine dependence | 6.00E-35 | 43 | ADRB2 SLC6A2 ADRB1 CA4 CA2 CHRM3 CACNA2D3 ADRA1A ADRA1D ADRA1B ADRA2A ADRA2C ADRA2B ADRB3 CACNA2D1 PTGS1 CACNA1D CACNA1C CACNA1F CACNA1S REN ACE AHR CA9 KCNMA1 AOC3 CHRM2 ACE2 NR3C2 KCNJ11 CACNA1B SLC12A3 SMPD1 CHRM1 EDNRB HTR1A CA1 HTR1B EDNRA CHRM5 CACNB4 CA12 TNNC2 |
| intrinsic cardiomyopathy | intrinsic cardiomyopathy | 8.00E-35 | 51 | TNNC1 CACNA1C ACE EDNRA CACNA1D KCNJ11 ADRA1B CACNA1S PTGS1 CACNA1F EDNRB SLC6A2 ADRB3 ADRB1 ADRB2 ADRA1A ADRA1D NR3C2 ACE2 REN AHR CACNA2D2 CA2 ADRA2A KCNMA1 CACNA1H PTGIR SLC12A1 KCNJ1 CACNA1B HTR1B CACNA2D3 CACNB4 ADRA2B CHRM3 SMPD1 PDE1B PDE1A HTR1A CHRM2 ADRA2C CA9 CACNB2 AOC3 CACNA2D1 CA1 CA4 CACNB1 SLC12A3 ATP1A1 AAAS |
| neurodegenerative disease | neurodegenerative disease | 1.00E-34 | 57 | CACNB4 ATP1A1 CHRM3 ADRB3 ADRA2B CACNA1B ADRA1B CHRM2 HTR1A SLC6A2 ADRB2 PTGS1 ADRB1 CHRM1 ADRA2A ADRA1D ADRA1A ADRA2C CACNA2D1 TNNC2 CACNA1F CACNA1S CACNA1D CACNA1C TNNC1 SMPD1 ACE CACNA2D3 KCNMA1 CA4 KCNJ11 REN CACNA2D2 CA2 AHR EDNRA NR3C2 EDNRB CACNB1 SLC12A1 CA1 P4HA1 PDE1A SLC12A3 CA12 KCNJ1 AOC3 HTR1B CACNA1H ACE2 AAAS PDE1B CA9 CHRM4 PTGIR CACNB2 CACNB3 |

| g3 | | | | |
| --- | --- | --- | --- | --- |
| Disease | Disease full name | Relevance (p-value) | Number of associated tc-genes3 | All tc-genes3 |
| cocaine dependence | cocaine dependence | 9.00E-39 | 23 | HTR2C CA2 CA4 SLC6A2 ADRB1 ADRB2 HTR1A ADRA1B ADRA1D ADRA1A ADRA2C ADRA2B ADRA2A CHRNB2 CHRNA4 ADRB3 HRH1 AGTR1 HTR1D CHRNA3 HTR1B CHRNB4 MMP9 |
| stress-related disorder | stress-related disorder | 2.00E-38 | 30 | HTR1A ADRB2 CA4 SLC6A2 HTR2C ADRA2B ADRB1 ADRA2C ADRA2A ADRA1D ADRA1B CA2 ADRA1A ADRB3 AGTR1 ACE CA1 CA3 HTR7 HTR1B MMP2 CHRNA3 CHRNA4 CHRNA2 CHRNB2 CHRNA10 CHRNA7 CHRNB4 MMP9 PTGFR |
| hypotension | hypotension | 8.00E-38 | 30 | ADRB2 ADRB1 SLC6A2 ADRA1B ADRA2A ADRA2C ADRA2B ADRA1A ADRB3 ADRA1D CA1 AGTR1 HRH2 HRH1 HTR1D CA2 CA4 ACE NISCH HTR7 HTR1A MMP9 HTR1B PTGIS BDKRB1 CA3 HTR2C MMP2 PTGIR CHRNA3 |
| neurotic disorder | neurotic disorder | 1.00E-36 | 29 | HTR1A ADRB2 CA4 SLC6A2 HTR2C ADRA2B ADRB1 ADRA1D ADRA2A ADRA1A CA2 HRH1 ADRA1B ADRA2C AGTR1 ADRB3 ACE CA1 CA3 CHRNA3 CHRNA4 HTR1B CHRNA7 CHRNB2 CHRNA2 CHRNB4 CHRNA10 MMP9 PTGFR |
| post-traumatic stress disorder | post-traumatic stress disorder | 1.00E-36 | 28 | HTR1A ADRB2 CA4 SLC6A2 HTR2C ADRA2B ADRB1 ADRA1B ADRA2A ADRA1D CA2 ADRA2C ADRA1A AGTR1 ADRB3 ACE CA1 CA3 HTR1B CHRNB2 CHRNA4 CHRNA2 CHRNB4 CHRNA3 CHRNA10 CHRNA7 MMP9 PTGFR |
| anxiety | anxiety | 2.00E-34 | 23 | HTR1A ADRA2A HTR2C HRH1 SLC6A2 ADRA2B ADRA2C ADRB3 ADRA1B ADRA1A ADRA1D ADRB1 ADRB2 HTR1B CA2 HTR1D HTR7 CA4 NISCH CHRNA4 CHRNB2 CA1 CA3 |
| pain | pain | 4.00E-34 | 37 | HRH1 ADRB2 ADRA2C ADRA2A ADRA1A HTR1B ADRB1 SLC6A2 HTR1D CA2 ADRB3 CA4 CHRNA4 ADRA1B ADRA1D ADRA2B CHRNB2 HTR2C PTGER3 CA1 PTGER1 BDKRB1 AGTR1 CA3 MMP9 MMP2 ACE HTR7 P2RY12 HTR1A HRH2 PTGIR PTGIS CHRNA7 PTGFR AKR1C3 CHRNA3 |
| anxiety disorder | anxiety disorder | 1.00E-33 | 36 | ADRB1 HTR1A HTR2C SLC6A2 ADRB2 CA4 ADRA2A ADRA2C ADRA2B HRH1 ADRA1B ADRA1D CA2 ADRA1A ADRB3 AGTR1 CA1 CA3 HTR7 HTR1B ACE HTR1D HRH2 CHRNA7 MMP9 CHRNA4 P2RY12 CHRNA3 MMP2 NISCH CHRNB2 AKR1C3 CHRNA2 CHRNA10 CHRNB4 PTGFR |
| drug-induced mental disorder | drug-induced mental disorder | 2.00E-33 | 38 | CHRNA4 HTR1A ADRB2 CHRNB2 SLC6A2 ADRA2A HTR2C ADRA2C ADRB1 CA4 ADRA2B ADRA1B ADRA1A ADRA1D CA2 HRH1 CHRNA3 ADRB3 CHRNB4 AGTR1 HTR1B MMP9 ACE HTR1D P2RY12 CA3 CHRNA7 MMP2 NISCH HTR7 CHRNA2 CA1 AKR1C3 HRH2 PTGIS LTA4H SFRP4 CHRNA10 |
| drug dependence | drug dependence | 2.00E-33 | 38 | CHRNA4 HTR1A ADRB2 CHRNB2 SLC6A2 ADRA2A HTR2C ADRA2C ADRB1 CA4 ADRA2B CA2 ADRA1A ADRA1D ADRA1B HRH1 CHRNA3 ADRB3 CHRNB4 AGTR1 HTR1B MMP9 ACE HTR1D P2RY12 CA3 CHRNA7 MMP2 NISCH HTR7 CHRNA2 CA1 AKR1C3 HRH2 PTGIS LTA4H SFRP4 CHRNA10 |
| bronchial disease | bronchial disease | 3.00E-33 | 37 | ADRB2 P2RY12 HRH1 ADRB1 ADRA2A ADRA1B ADRB3 SLC6A2 ADRA1A ADRA1D CHRNA4 CHRNB2 ADRA2B ADRA2C CHRNA3 CHRNA7 CHRNB4 MMP9 MMP2 HTR1A HTR2C PTGIR LTA4H ACE HRH2 SFRP4 AGTR1 BDKRB1 CHRNA2 PTGER3 CA1 AKR1C3 HTR1B HTR7 PTGIS CA4 PTGFR |
| orthostatic hypotension | orthostatic hypotension | 4.00E-33 | 20 | ADRB2 ADRB1 ADRA2A ADRA2C ADRA2B ADRA1A SLC6A2 ADRB3 ADRA1B ADRA1D HRH1 AGTR1 HTR1D CA1 CA2 CA4 HRH2 HTR1A ACE HTR2C |
| cognitive impairment | cognitive impairment | 5.00E-33 | 35 | CA2 CA4 HTR1A HTR2C HRH1 ADRA2A ADRA1B SLC6A2 ADRA1D ADRA2B ADRA1A ADRA2C CHRNA7 HTR7 HTR1B HTR1D ADRB1 ADRB3 ADRB2 AGTR1 CHRNA4 CHRNB2 MMP2 MMP9 CA1 ACE CA3 PTGIS BDKRB1 NISCH HRH2 P2RY12 PTGFR CHRNB4 CHRNA3 |
| Abnormality of the respiratory... | Abnormality of the respiratory system | 2.00E-32 | 32 | ADRB2 HRH1 ADRB1 ADRB3 ADRA1B ADRA2C ADRA2A ADRA2B ADRA1A SLC6A2 ADRA1D HRH2 CHRNA7 MMP9 ACE CA1 P2RY12 MMP2 HTR1A AGTR1 HTR7 LTA4H CA3 HTR2C NISCH CHRNA3 PTGER3 HTR1B HTR1D PTGIS PTGIR CHRNB4 |
| abnormality of higher mental f... | abnormality of higher mental function | 4.00E-32 | 35 | CA2 CA4 HTR1A HTR2C HRH1 ADRA2A ADRA2C ADRA1D SLC6A2 ADRA1B ADRA1A ADRA2B CHRNA7 HTR7 HTR1B HTR1D ADRB1 ADRB3 ADRB2 AGTR1 CHRNA4 CHRNB2 MMP2 MMP9 CA1 ACE CA3 PTGIS P2RY12 BDKRB1 NISCH HRH2 PTGFR CHRNB4 CHRNA3 |
| coronary heart disease | coronary heart disease | 5.00E-32 | 36 | ADRB2 P2RY12 ACE AGTR1 ADRB1 ADRB3 HRH2 ADRA1A PTGER1 ADRA1D ADRA1B CHRNA4 CHRNB2 CHRNB4 SFRP4 ADRA2A ADRA2B ADRA2C CHRNA7 MMP9 MMP2 HTR1B PTGIS SLC6A2 CA1 LTA4H HTR2C NISCH CA3 CA4 HTR1D HRH1 CA2 BDKRB1 AKR1C3 CHRNA3 |
| upper respiratory tract diseas... | upper respiratory tract disease | 6.00E-32 | 40 | ADRB2 CA2 CA1 CHRNA3 HRH1 ACE CA4 ADRB3 ADRA2C AGTR1 ADRB1 ADRA2A PTGIR ADRA1B SLC6A2 ADRA2B ADRA1D ADRA1A CHRNB4 MMP9 SFRP4 P2RY12 CHRNA7 CHRNB2 CHRNA4 LTA4H HRH2 MMP2 HTR2C HTR1A CA3 HTR7 BDKRB1 HTR1B PTGER3 PTGIS CHRNA2 NISCH HTR1D AKR1C3 |
| disease of central nervous sys... | disease of central nervous system or retinal vasculature | 2.00E-31 | 41 | ACE P2RY12 SLC6A2 AGTR1 ADRB1 ADRA2A PTGER1 ADRA2B ADRA2C PTGFR CA1 ADRA1B ADRA1A ADRA1D CA2 CA4 MMP9 HTR1A CHRNB4 ADRB2 HTR1B MMP2 ADRB3 CHRNA7 CHRNA3 CHRNA4 CHRNB2 HTR7 PTGIR PTGER3 BDKRB1 HRH1 LTA4H CA3 PTGIS HTR2C HRH2 NISCH SFRP4 HTR1D AAAS |
| tracheal disease | tracheal disease | 2.00E-31 | 38 | ADRB2 CA2 CA1 CHRNA3 ACE CA4 ADRB3 ADRA2C AGTR1 ADRB1 ADRA2A PTGIR ADRA1B ADRA2B ADRA1A ADRA1D CHRNB4 MMP9 CHRNA7 P2RY12 CHRNB2 CHRNA4 LTA4H HTR2C MMP2 HRH2 HTR1A CA3 HTR1B BDKRB1 PTGER3 PTGIS SFRP4 CHRNA2 SLC6A2 NISCH HTR1D HTR7 |
| chronic obstructive pulmonary ... | chronic obstructive pulmonary disease | 2.00E-31 | 38 | ADRB2 CA2 CA1 CHRNA3 ACE CA4 ADRB3 ADRA2C AGTR1 ADRB1 ADRA2A PTGIR ADRA1B ADRA2B ADRA1D ADRA1A CHRNB4 MMP9 CHRNA7 P2RY12 CHRNA4 CHRNB2 LTA4H HTR2C MMP2 HRH2 HTR1A CA3 HTR1B BDKRB1 PTGER3 PTGIS SFRP4 CHRNA2 SLC6A2 NISCH HTR1D HTR7 |

| g4 | | | | |
| --- | --- | --- | --- | --- |
| Disease | Disease full name | Relevance (p-value) | Number of associated tc-genes4 | All tc-genes4 |
| Renal insufficiency | Renal insufficiency | 7.00E-44 | 22 | KCNH2 CACNB4 CACNG1 KCNH7 KCNH6 CACNA1H CACNA1G CACNB2 CACNA1D CACNA1F CACNA1C CACNA1I CACNB3 CACNA1S CACNB1 ADRA2B ADRA2A ADRA1B ADRB1 ADRA1D ADRB2 ADRA1A |
| abnormal renal physiology | abnormal renal physiology | 6.00E-41 | 25 | ADRA2B ADRA2A PDE5A KCNH2 CACNA1D CACNB4 CACNA1F CACNB2 CACNA1G CACNG1 CACNB3 CACNA1C KCNH7 CACNB1 CACNA1I KCNH6 CACNA1H CACNA1S ADRB1 ADRB2 ADRA1D ADRA1A ADRA1B ACE EDNRB |
| abnormality of the urinary sys... | abnormality of the urinary system physiology | 7.00E-41 | 25 | ADRA2A ADRA1D ADRA1B ADRA1A ADRA2B PDE5A KCNH2 CACNB1 CACNA1D CACNB3 CACNA1H CACNA1F KCNH6 KCNH7 CACNA1C CACNB4 CACNG1 CACNA1G CACNB2 CACNA1S CACNA1I ADRB1 ADRB2 ACE EDNRB |
| anxiety | anxiety | 1.00E-30 | 19 | ADRA2A ADRA2B ADRA1B ADRA1A ADRA1D ADRB1 ADRB2 CACNG1 CACNB4 CACNB1 CACNA1C CACNA1G CACNA1F CACNB3 CACNB2 CACNA1H CACNA1S CACNA1D CACNA1I |
| anxiety disorder | anxiety disorder | 5.00E-29 | 28 | ADRB1 CACNA1C ADRB2 ADRA2A ADRA2B CACNB2 ADRA1B ADRA1D CACNA1I CACNA1F CACNB3 CACNG1 CACNA1H CACNB4 ADRA1A CACNA1D CACNA1S CACNA1G CACNB1 EDNRB ACE GUCY1A2 SLC18A1 SLC18A2 PDE5A EDNRA KCNH7 KCNH2 |
| Ischemic stroke | Ischemic stroke | 7.00E-26 | 15 | CACNA1C CACNA1F CACNA1D CACNA1S KCNH6 KCNH2 KCNH7 ADRA1A ADRA1B ADRA1D ADRA2A ADRB2 ADRB1 ADRA2B PDE5A |
| insomnia | insomnia | 3.00E-25 | 17 | ADRA2A ADRA2B CACNA1D CACNA1C CACNA1I CACNG1 CACNA1S CACNA1F CACNB2 CACNA1H CACNB1 CACNB3 CACNB4 CACNA1G SLC18A2 ACE ADRB1 |
| Sleep Disorder | Sleep Disorder | 1.00E-24 | 22 | SLC18A2 ADRA2A ADRA2B CACNA1D CACNA1C CACNA1G CACNA1F CACNB4 CACNB2 CACNB1 CACNA1S CACNA1H CACNA1I CACNG1 CACNB3 ADRB2 ADRB1 KCNH2 KCNH6 KCNH7 ACE EDNRA |
| cardioectodermal syndrome | cardioectodermal syndrome | 1.00E-24 | 22 | SLC18A2 ADRA2A ADRA2B CACNA1D CACNA1C CACNB2 CACNA1S CACNA1F CACNA1I CACNB4 CACNB1 CACNG1 CACNA1H CACNA1G CACNB3 ADRB2 ADRB1 KCNH2 KCNH6 KCNH7 ACE EDNRA |
| behavioral abnormality | behavioral abnormality | 2.00E-24 | 24 | ADRA2A ADRA2B ADRA1B ADRA1A ADRA1D ADRB2 ADRB1 CACNA1G CACNA1C CACNB2 CACNB1 CACNA1S CACNB3 CACNA1F CACNB4 CACNA1D CACNA1H CACNA1I CACNG1 PDE5A ACE SLC18A2 EDNRA EDNRB |
| Cerebral ischemia | Cerebral ischemia | 2.00E-23 | 15 | CACNA1F CACNA1C CACNA1S CACNA1D KCNH7 KCNH2 KCNH6 ADRA1A ADRA1B ADRA1D ADRA2A ADRA2B ADRB2 ADRB1 PDE5A |
| migraine disorder | migraine disorder | 6.00E-23 | 19 | ADRA1B ADRB2 ADRA2B ADRB1 ADRA1A ADRA1D ADRA2A PDE5A CACNA1G CACNA1H CACNA1I EDNRA ACE EDNRB CACNA1C CACNB2 CACNB4 CACNA1F CACNA1S |
| hypnic headache (disease) | hypnic headache (disease) | 6.00E-23 | 19 | ADRA1B ADRB2 ADRA2B ADRB1 ADRA1A ADRA1D ADRA2A PDE5A CACNA1G CACNA1H CACNA1I EDNRA ACE EDNRB CACNA1C CACNB2 CACNB4 CACNA1F CACNA1S |
| primary hypertension | primary hypertension | 6.00E-22 | 16 | ACE ADRB1 CACNA1C CACNA1D CACNA1F CACNA1S ADRB2 ADRA1B ADRA1D ADRA1A PDE5A CACNB2 EDNRA ADRA2B KCNH2 EDNRB |
| intracerebral hemorrhage | intracerebral hemorrhage | 1.00E-21 | 16 | ACE CACNA1C CACNA1D CACNA1S CACNA1F ADRB2 ADRA2A ADRA1D ADRA1A ADRB1 ADRA1B ADRA2B EDNRA EDNRB SLC18A2 CACNB2 |
| ventricular tachycardia | ventricular tachycardia | 1.00E-21 | 14 | KCNH2 ADRB1 ADRB2 ACE ADRA2A ADRA1B ADRA1A ADRA1D ADRA2B CACNA1D SLC18A2 CACNA1C EDNRA CACNA1S |
| sudden cardiac arrest | sudden cardiac arrest | 2.00E-21 | 14 | ADRA1B ADRA2A KCNH2 ADRB2 ADRA1D ADRB1 ADRA1A ADRA2B CACNA1C CACNA1H ACE CACNA1D CACNA1G CACNB2 |
| osteoarthritis knee | osteoarthritis knee | 2.00E-21 | 12 | ADRA2A ADRA1B ADRA1D ADRA1A ADRB1 ADRA2B ADRB2 CACNA1C CACNA1F CACNA1D CACNA1S ACE |
| headache disorder | headache disorder | 3.00E-21 | 20 | ADRA1B ADRB2 ADRA2B ADRB1 ADRA1A ADRA2A ADRA1D CACNA1F CACNA1G CACNB4 SLC18A2 PDE5A CACNA1I CACNA1H EDNRA ACE EDNRB CACNA1C CACNB2 CACNA1S |
| heart disease | heart disease | 5.00E-21 | 31 | CACNA1C KCNH2 CACNA1D EDNRA CACNB2 ACE SLC18A2 ADRA2A ADRA1B CACNA1S KCNH7 EDNRB GUCY1A2 CACNA1F ADRA2B ADRB2 PDE5A ADRB1 ADRA1A ADRA1D KCNH6 SLC18A1 CACNA1G CACNA1H CACNB4 CACNB1 CACNB3 CACNG1 CACNA1I PDE11A BIRC5 |

| g5 | | | | |
| --- | --- | --- | --- | --- |
| Disease | Disease full name | Relevance (p-value) | Number of associated tc-genes5 | All tc-genes5 |
| abnormal renal physiology | abnormal renal physiology | 4.00E-44 | 30 | SLC12A1 ADRA2B ADRA2A ADRA2C DRD1 PDE1A TNNC1 PDE1B KCNH2 CACNA1A CACNA1D CACNA2D2 CACNA2D1 CACNB2 CACNG1 CACNA1C KCNQ1 CACNA1H CACNA1S ADRB1 ADRB2 ADRA1D ADRA1A ADRA1B CA2 ACE HIF1A GJA1 VEGFA SLC12A3 |
| abnormality of the urinary sys... | abnormality of the urinary system physiology | 4.00E-44 | 30 | SLC12A1 ADRA2A ADRA1D ADRA1B ADRA1A ADRA2C ADRA2B DRD1 PDE1A PDE1B TNNC1 KCNH2 KCNQ1 CACNA1A CACNA1D CACNA1H CACNA2D2 CACNA1C CACNA2D1 CACNG1 CACNB2 CACNA1S ADRB1 ADRB2 CA2 ACE HIF1A GJA1 VEGFA SLC12A3 |
| heart failure | heart failure | 4.00E-37 | 37 | ACE ADRB1 ADRA2C AGTR1 ADRB2 SLC12A1 ADRA1D CA4 KCNH2 P2RY12 ADRA2A CA2 PTGIR SLC12A3 CA1 ADRA1B ADRA2B CACNA1S CACNA1D TNNC1 NDUFC2 CACNA1C ADRA1A ATP1A1 VEGFA GJA1 HIF1A SELE VCAM1 PPARD PDE1A CACNB2 NPPB CALM1 SFRP4 DRD1 KCNQ1 |
| heart conduction disease | heart conduction disease | 6.00E-36 | 36 | KCNQ1 CACNA1C KCNH2 CACNA1D CALM1 CACNB2 AGTR1 ADRA1B ADRA2A CACNA1S ADRA1A ADRB1 P2RY12 ADRB2 ADRA2B ADRA2C NDUFC2 ATP1A1 ADRA1D ACE GJA1 VEGFA CACNA2D2 CA2 CACNA1H NPPB DRD5 CA1 CA4 VCAM1 SLC12A3 SELE CACNA2D1 HIF1A PPARD CACNA1A |
| anxiety disorder | anxiety disorder | 8.00E-36 | 38 | ADRB1 CACNA1C ADRB2 CA4 ADRA2A ADRA2C ADRA2B CACNB2 CACNA1A ADRA1B ADRA1D CACNG1 CA2 CACNA1H CACNA2D2 ADRA1A CACNA1D CACNA1S CACNA2D1 VEGFA AGTR1 CA1 ACE NDUFC2 PPARD DRD1 GJA1 P2RY12 ATP1A1 HIF1A PDE1B KCNQ1 PDE1A VCAM1 DRD5 SELE CALM1 KCNH2 |
| congestive heart failure | congestive heart failure | 7.00E-35 | 30 | ACE ADRB1 SLC12A1 ADRB2 AGTR1 ADRA1D PTGIR P2RY12 SLC12A3 CACNA1S ADRA1A ADRA1B TNNC1 NDUFC2 CACNA1D ATP1A1 CACNA1C KCNH2 VEGFA GJA1 HIF1A PPARD PDE1A SELE ADRA2C ADRA2A NPPB CA1 VCAM1 KCNQ1 |
| cardiac arrest | cardiac arrest | 9.00E-35 | 27 | KCNH2 ADRA2A ADRA1B ADRB2 ADRA1A ADRA1D ADRB1 ADRA2B ADRA2C GJA1 AGTR1 VEGFA CACNA1C PTGIR CACNA1H NPPB ACE CACNA1D KCNQ1 CA1 HIF1A P2RY12 VCAM1 CALM1 SELE CA4 CACNB2 |
| cardiac rhythm disease | cardiac rhythm disease | 2.00E-34 | 35 | KCNQ1 CACNA1C KCNH2 CACNA1D CALM1 CACNB2 AGTR1 ADRA1B ADRA2A CACNA1S ADRA1A ADRB1 P2RY12 ADRB2 ADRA2B ADRA2C NDUFC2 ADRA1D ATP1A1 ACE GJA1 VEGFA CACNA2D2 CA2 NPPB CACNA1H CA1 CA4 VCAM1 SLC12A3 SELE CACNA2D1 HIF1A PPARD CACNA1A |
| primary hypertension | primary hypertension | 2.00E-34 | 25 | ACE ADRB1 AGTR1 SLC12A3 CACNA1C CACNA1D CACNA1S ADRB2 ADRA1B ADRA1D ADRA1A ATP1A1 SELE VEGFA DRD1 CACNB2 PPARD ADRA2B DRD5 SLC12A1 KCNH2 GJA1 VCAM1 CA1 NPPB |
| stroke | stroke | 3.00E-34 | 37 | P2RY12 ACE AGTR1 ADRB1 CACNA1C PDE1B CACNA1S CACNA1D PDE1A ADRA1A ADRA1B ADRA1D KCNQ1 KCNH2 CACNB2 ADRB2 VEGFA ADRA2B ADRA2C ADRA2A CA1 CA4 CA2 TNNC1 VCAM1 HIF1A SELE GJA1 PPARD NDUFC2 CALM1 PTGIR DRD1 NPPB SFRP4 CACNA1A SLC12A3 |
| hypotension | hypotension | 3.00E-33 | 28 | ADRB2 ADRB1 ADRA1B ADRA2A ADRA2C ADRA2B ADRA1A ADRA1D CA1 AGTR1 CA2 CA4 CACNA1C CACNA1D CACNA1S VEGFA ACE HIF1A GJA1 SLC12A3 SLC12A1 SELE NPPB PTGIR DRD1 KCNH2 CACNB2 KCNQ1 |
| cerebrovascular disorder | cerebrovascular disorder | 5.00E-33 | 40 | ACE P2RY12 AGTR1 ADRB1 ADRA2A CACNA1C ADRA2B ADRA2C CACNA1D CACNA1S PDE1B PDE1A CACNA2D1 CA1 ADRA1A ADRA1B ADRA1D CA4 CA2 KCNQ1 SLC12A3 KCNH2 VEGFA CACNB2 ADRB2 TNNC1 HIF1A DRD1 PTGIR DRD5 VCAM1 GJA1 SELE PPARD SLC12A1 NDUFC2 CALM1 CACNA1A NPPB SFRP4 |
| arterial disorder | arterial disorder | 1.00E-32 | 44 | CACNA1D SLC12A1 ACE AGTR1 ADRB1 CACNA1C SLC12A3 CA2 ADRA1D ADRB2 DRD1 P2RY12 ADRA2B ADRA1B PTGIR ADRA2C PDE1A ADRA2A NDUFC2 PDE1B ADRA1A TNNC1 CACNA1S CA1 CA4 VEGFA CACNB2 DRD5 KCNQ1 CACNA2D2 SELE HIF1A SFRP4 CACNA1A PPARD ATP1A1 GJA1 VCAM1 KCNH2 NPPB CACNA1H FXYD2 CACNA2D1 CALM1 |
| hypertension | hypertension | 1.00E-32 | 41 | CACNA1D SLC12A1 ACE AGTR1 ADRB1 CACNA1C SLC12A3 CA2 ADRA1D ADRB2 ADRA2B ADRA1B PTGIR ADRA2C P2RY12 ADRA2A ADRA1A NDUFC2 CACNA1S CA1 CA4 CACNB2 VEGFA DRD5 HIF1A PPARD ATP1A1 GJA1 DRD1 PDE1A SELE PDE1B VCAM1 KCNQ1 NPPB CACNA1A CACNA1H SFRP4 CACNA2D1 FXYD2 KCNH2 |
| congenital heart disease | congenital heart disease | 2.00E-32 | 34 | KCNQ1 CACNA1C GJA1 KCNH2 CALM1 VEGFA ADRA2A ACE CACNA1D HIF1A ADRA2C ADRA2B ADRA1B CACNA1S ADRA1D PTGIR ADRA1A TNNC1 AGTR1 NPPB CACNA1H KCNJ4 CA4 CA2 CA1 PPARD P2RY12 SLC12A3 SELE VCAM1 ADRB2 ADRB1 CACNA1A CACNB2 |
| congenital anomaly of cardiova... | congenital anomaly of cardiovascular system | 3.00E-32 | 34 | KCNQ1 CACNA1C GJA1 KCNH2 CALM1 VEGFA ADRA2A ACE CACNA1D HIF1A ADRA2B ADRA2C PTGIR ADRA1B CACNA1S ADRA1A ADRA1D TNNC1 AGTR1 NPPB CACNA1H KCNJ4 CA4 CA2 CA1 PPARD P2RY12 SLC12A3 SELE VCAM1 ADRB2 ADRB1 CACNA1A CACNB2 |
| anxiety | anxiety | 7.00E-32 | 22 | ADRA2A CACNA2D1 ADRA2B ADRA2C ADRA1B ADRA1A ADRA1D ADRB1 ADRB2 CACNG1 CACNA1C CACNB2 CACNA1A CACNA2D2 CACNA1H CACNA1S CACNA1D CA2 CA4 DRD5 DRD1 CA1 |
| Renal insufficiency | Renal insufficiency | 1.00E-31 | 19 | KCNH2 KCNQ1 CACNG1 CACNA1H CACNB2 CACNA2D1 CACNA2D2 CACNA1A CACNA1D CACNA1C CACNA1S ADRA2B ADRA2A ADRA1B ADRA2C ADRB1 ADRA1D ADRB2 ADRA1A |
| myocardial disorder | myocardial disorder | 2.00E-31 | 35 | ACE CACNA1D P2RY12 ADRB2 PPARD AGTR1 ADRB1 KCNH2 ADRA1D SLC12A3 CACNA1C ADRA1A CACNA1S ADRA1B KCNQ1 PDE1B SLC12A1 PDE1A TNNC1 VEGFA ADRA2A GJA1 HIF1A VCAM1 SELE ADRA2B ADRA2C DRD1 NDUFC2 CA1 CACNA2D2 PTGIR SFRP4 CA4 NPPB |
| disease of central nervous sys... | disease of central nervous system or retinal vasculature | 4.00E-31 | 42 | CACNA1A VEGFA ACE P2RY12 CACNA1S AGTR1 ADRB1 ADRA2A CACNA1C ADRA2B ADRA2C PDE1B CACNA2D1 PDE1A CACNA1D CA1 ADRA1B ADRA1A ADRA1D SELE CA2 CA4 KCNQ1 SLC12A3 KCNH2 CACNB2 ADRB2 CACNA2D2 DRD5 TNNC1 HIF1A GJA1 DRD1 PTGIR ATP1A1 VCAM1 SLC12A1 NDUFC2 PPARD CALM1 NPPB SFRP4 |

| g6 | | | | |
| --- | --- | --- | --- | --- |
| Disease | Disease full name | Relevance (p-value) | Number of associated tc-genes6 | All tc-genes6 |
| orthostatic hypotension | orthostatic hypotension | 1.00E-21 | 11 | ADRB2 ADRB1 ADRA2A ADRA2C ADRA2B SLC6A2 ADRB3 MAOB DDC MAOA REN |
| fibromyalgia | fibromyalgia | 1.00E-21 | 11 | SLC6A2 ADRA2A ADRA2B ADRA2C ADRB2 ADRB1 ADRB3 DDC REN MAOA MAOB |
| substance withdrawal syndrome | substance withdrawal syndrome | 3.00E-19 | 11 | ADRA2A ADRA2C ADRA2B REN ADRB2 MAOA DDC MAOB NPR1 SLC6A2 ADRB1 |
| Headache | Headache | 7.00E-19 | 12 | ADRB1 SLC6A2 ADRB2 ADRA2C ADRA2A ADRA2B NPR1 MAOA REN MAOB DDC ATP1A1 |
| hypotension | hypotension | 9.00E-19 | 13 | ADRB2 ADRB1 SLC6A2 ADRA2A ADRA2C ADRA2B ADRB3 MAOB DDC MAOA REN NPR1 KCNJ1 |
| multiple system atrophy | multiple system atrophy | 1.00E-18 | 11 | DDC MAOB ADRB1 ADRB2 ADRA2C ADRA2A ADRA2B ADRB3 TH SLC6A2 REN |
| cocaine dependence | cocaine dependence | 1.00E-18 | 10 | SLC6A2 MAOB ADRB1 ADRB2 DDC ADRA2C ADRA2B ADRA2A ADRB3 MAOA |
| Bronchiolitis | Bronchiolitis | 6.00E-18 | 6 | ADRA2A ADRA2C ADRB2 ADRB1 ADRA2B ADRB3 |
| Atrophy | Atrophy | 8.00E-18 | 11 | DDC MAOB ADRB1 ADRB2 ADRA2C TH ADRA2B ADRB3 ADRA2A SLC6A2 REN |
| adrenal gland disease | adrenal gland disease | 1.00E-17 | 16 | ADRA2A ADRA2B ADRA2C ATP1A1 REN NPR1 ABCC9 KCNJ1 SLC6A2 DDC ADRB3 TH ADRB2 MAOA ADRB1 MAOB |
| cardiac arrhythmia | cardiac arrhythmia | 2.00E-17 | 15 | ABCC9 ADRA2A SLC6A2 ADRB2 ADRB1 ADRB3 ADRA2B ADRA2C ATP1A1 REN TH DDC NPR1 MAOB MAOA |
| primary orthostatic hypotensio... | primary orthostatic hypotension | 3.00E-17 | 11 | SLC6A2 DDC ADRB2 TH MAOB ADRB1 ADRA2B ADRA2C ADRA2A ADRB3 REN |
| Abnormal cardiovascular system... | Abnormal cardiovascular system physiology | 3.00E-17 | 12 | ADRB1 REN ADRB2 SLC6A2 ABCC9 ADRB3 NPR1 ADRA2C ADRA2A KCNJ1 DDC MAOA |
| autonomic nervous system disea... | autonomic nervous system disease | 4.00E-17 | 13 | SLC6A2 ADRA2B ADRA2A ADRA2C MAOA REN TH DDC ADRB2 ADRB1 MAOB ATP1A1 NPR1 |
| anxiety | anxiety | 9.00E-17 | 10 | ADRA2A SLC6A2 ADRA2B ADRA2C ADRB3 ADRB1 ADRB2 MAOB MAOA REN |
| Abnormality of cardiovascular ... | Abnormality of cardiovascular system electrophysiology | 9.00E-17 | 11 | ADRB1 ADRB2 REN SLC6A2 ABCC9 ADRB3 NPR1 ADRA2A ADRA2C MAOA DDC |
| adrenal gland pheochromocytoma | adrenal gland pheochromocytoma | 1.00E-16 | 12 | ADRA2B ADRA2C ADRA2A REN SLC6A2 TH DDC ADRB2 MAOA ADRB1 MAOB NPR1 |
| sympathetic paraganglioma | sympathetic paraganglioma | 1.00E-16 | 12 | ADRA2B ADRA2C ADRA2A REN SLC6A2 TH DDC ADRB2 MAOA ADRB1 MAOB NPR1 |
| adrenal medulla neoplasm | adrenal medulla neoplasm | 2.00E-16 | 12 | ADRA2B ADRA2A ADRA2C REN SLC6A2 TH DDC ADRB2 MAOA ADRB1 MAOB NPR1 |
| croup | croup | 2.00E-16 | 6 | ADRB3 ADRA2C ADRA2A ADRB2 ADRB1 ADRA2B |

**Table S6.** List of gold-standard pairwise combinations of the AHs

| S. No. | DrugBank_ID_A | Name_A | Group_A | DrugBank_ID_B | Name_B | Group_B |
| --- | --- | --- | --- | --- | --- | --- |
| 1 | DB00264 | Metoprolol | g6 | DB00381 | Amlodipine | g2 |
| 2 | DB00381 | Amlodipine | g2 | DB00335 | Atenolol | g6 |
| 3 | DB00335 | Atenolol | g6 | DB00436 | Bendroflumethiazide | g2 |
| 4 | DB01193 | Acebutolol | g6 | DB00999 | Hydrochlorothiazide | g2 |
| 5 | DB00381 | Amlodipine | g2 | DB00999 | Hydrochlorothiazide | g2 |
| 6 | DB00335 | Atenolol | g6 | DB00999 | Hydrochlorothiazide | g2 |
| 7 | DB00612 | Bisoprolol | g6 | DB00999 | Hydrochlorothiazide | g2 |
| 8 | DB01197 | Captopril | g6 | DB00999 | Hydrochlorothiazide | g2 |
| 9 | DB01275 | Hydralazine | g2 | DB00999 | Hydrochlorothiazide | g2 |
| 10 | DB00722 | Lisinopril | g2 | DB00999 | Hydrochlorothiazide | g2 |
| 11 | DB00968 | Methyldopa | g6 | DB00999 | Hydrochlorothiazide | g2 |
| 12 | DB00691 | Moexipril | g2 | DB00999 | Hydrochlorothiazide | g2 |
| 13 | DB00966 | Telmisartan | g1 | DB00999 | Hydrochlorothiazide | g2 |
| 14 | DB04861 | Nebivolol | g4 | DB00808 | Indapamide | g5 |
| 15 | DB00381 | Amlodipine | g2 | DB01029 | Irbesartan | g1 |
| 16 | DB00999 | Hydrochlorothiazide | g2 | DB01029 | Irbesartan | g1 |
| 17 | DB01023 | Felodipine | g2 | DB00264 | Metoprolol | g6 |
| 18 | DB00999 | Hydrochlorothiazide | g2 | DB00264 | Metoprolol | g6 |
| 19 | DB00436 | Bendroflumethiazide | g2 | DB01203 | Nadolol | g2 |
| 20 | DB00381 | Amlodipine | g2 | DB00966 | Telmisartan | g1 |
| 21 | DB00999 | Hydrochlorothiazide | g2 | DB00373 | Timolol | g3 |
